# Predicting Multiple Sclerosis Outcomes During the COVID-19 Stay-at-home Period: Observational Study Using Passively Sensed Behaviors and Digital Phenotyping

**DOI:** 10.2196/38495

**Published:** 2022-08-24

**Authors:** Prerna Chikersal, Shruthi Venkatesh, Karman Masown, Elizabeth Walker, Danyal Quraishi, Anind Dey, Mayank Goel, Zongqi Xia

**Affiliations:** 1 School of Computer Science Carnegie Mellon University Pittsburgh, PA United States; 2 Department of Neurology University of Pittsburgh Pittsburgh, PA United States; 3 Information School University of Washington, Seattle Seattle, WA United States

**Keywords:** mobile sensing, sensor, sensing, mobile health, mHealth, algorithm, multiple sclerosis, disability, mental health, depression, sleep, fatigue, tiredness, predict, machine learning, feature selection, neurological disorder, COVID-19, isolation, behavior change, health outcome, fitness, movement, physical activity, exercise, tracker, digital phenotyping

## Abstract

**Background:**

The COVID-19 pandemic has broad negative impact on the physical and mental health of people with chronic neurological disorders such as multiple sclerosis (MS).

**Objective:**

We presented a machine learning approach leveraging passive sensor data from smartphones and fitness trackers of people with MS to predict their health outcomes in a natural experiment during a state-mandated stay-at-home period due to a global pandemic.

**Methods:**

First, we extracted features that capture behavior changes due to the stay-at-home order. Then, we adapted and applied an existing algorithm to these behavior-change features to predict the presence of depression, high global MS symptom burden, severe fatigue, and poor sleep quality during the stay-at-home period.

**Results:**

Using data collected between November 2019 and May 2020, the algorithm detected depression with an accuracy of 82.5% (65% improvement over baseline; F_1_-score: 0.84), high global MS symptom burden with an accuracy of 90% (39% improvement over baseline; F_1_-score: 0.93), severe fatigue with an accuracy of 75.5% (22% improvement over baseline; F_1_-score: 0.80), and poor sleep quality with an accuracy of 84% (28% improvement over baseline; F_1_-score: 0.84).

**Conclusions:**

Our approach could help clinicians better triage patients with MS and potentially other chronic neurological disorders for interventions and aid patient self-monitoring in their own environment, particularly during extraordinarily stressful circumstances such as pandemics, which would cause drastic behavior changes.

## Introduction

The COVID-19 pandemic and the ensuing response (eg, lockdown and social distancing) have broad negative impacts on physical and mental health worldwide [1-7]. The effect is more pronounced for people with chronic neurological diseases such as multiple sclerosis (MS) [8-10]. People with MS have a significantly higher burden of mental health comorbidities than the general population. Moreover, people with MS have a 50% lifetime prevalence of depression, 2-3 times higher than the general population [11-13]. Given its association with higher disability and mortality, depression is a major comorbidity that lowers the quality of life [11,14-20]. Further, people with MS have greater COVID-19 risk due to certain immune disease-modifying therapies as well as their physical disability, and many have experienced drastic change in their neurological care due to the pandemic [21]. Concerns for COVID-19, coupled with decreased social support and health care access during the pandemic, have contributed to even higher stress and depression in people with MS [10,22-24].

During the pandemic, digital technologies have become invaluable for supporting social interaction, health care access, and health monitoring. Digital health tools can also measure an individual’s mental health profile based on passive (noninvasive) tracking. Given the complexity and heterogeneity of real-world behaviors, models that leverage different aspects of an individual’s daily behaviors are necessary to accurately predict mental health status. Relevant to depression in people with MS, clinicians could use this digital passive sensing approach to potentially identify patients who require urgent health interventions.

Past research has leveraged passively generated data from personal digital devices (eg, smartphones and fitness trackers) to capture human behavior and predict health outcomes. This moment-by-moment, in situ quantification of the individual-level human phenotype using data from personal digital devices is known as digital phenotyping [25]. Previous works using passively sensed smartphone and wearable data to predict physical disability and fatigue in people with MS have been exploratory in assessing the feasibility of data collection and the preliminary association between sensed behaviors and outcomes [26-28]. However, the clinical applicability of digital phenotyping to inform clinical outcomes in people with MS in the real world has not yet been established.

Here, we present a machine learning approach leveraging data from the smartphones and fitness trackers of people with MS to predict their health outcomes during a mandatory stay-at-home period of the pandemic. Building on an existing analytical pipeline [29], we quantified behavior changes during the stay-at-home period when compared to the preceding period and used the changes to predict the presence of patient-reported outcomes of depression, neurological disability, fatigue, and poor sleep quality during the stay-at-home period. This study is different from prior studies in that it examines the clinical utility of digital phenotyping with passive sensors for predicting health outcomes during the early wave of the COVID-19 pandemic in a unique natural experiment. The study has relevance for predicting the health outcomes of patients with chronic and complex conditions beyond MS during major stressful scenarios (eg, pandemics and natural disasters) that could considerably alter behaviors.

## Methods

### Overview

This study was part of a larger study that aimed to examine the clinical utility of passive sensors on smartphones and fitness trackers in predicting clinically relevant outcomes in people with MS. Data collection from participants in this larger study occurred between November 2019 and January 2021. Because data collection for 56 participants spanned the locally mandated stay-at-home period in response to the COVID-19 pandemic, we used this unique natural experiment to test the hypothesis whether machine learning models leveraging passive sensor data can predict the health outcomes of people with a chronic neurological disorder (ie, people with MS) during major stressful scenarios.

To briefly summarize our approach, we used data from 3 sensors in the participants’ smartphones (calls, location, and screen activity) and 3 sensors in the participants’ fitness trackers (heart rate, sleep, and steps) to predict patient-reported outcomes of depression, global MS symptom burden, fatigue, and sleep quality during the COVID-19 stay-at-home period. We computed behavioral features from these 6 sensors before and during the stay-at-home period and took the difference as a measure of behavior change resulting from the stay-at-home mandate. We then used changes in behavioral features to predict the outcomes.

All methods were performed in accordance with the institutional review board guideline and institutional regulation.

### Participants

The study included adults 18 years or older with a neurologist-confirmed MS diagnosis who owned a smartphone (Android or iOS) and enrolled in the Prospective Investigation of Multiple Sclerosis in the Three Rivers Region study, a clinic-based natural history study at the University of Pittsburgh Medical Center [21,30-34].

### Ethical Considerations

The institutional review boards of University of Pittsburgh (STUDY19080007) and Carnegie Mellon University (STUDY2019_00000037) approved the study. All participants provided written informed consent.

### Study Design

The participants downloaded a mobile app to capture sensor data from their own smartphones and additionally received a Fitbit Inspire HR (Fitbit Inc) to track steps, heart rate, and sleep. Data were continuously collected from smartphone and Fitbit sensors of 56 participants during the study period (16 November 2019 to 15 May 2020, including the local stay-at-home period).

All 56 (100%) participants completed data collection for a predefined period of 12 weeks while 39 (70%) agreed to extend data collection for an additional 12 weeks (for a total of 24 weeks). Moreover, 6 (11%) participants who did not have sufficient data during the period before the stay-at-home mandate were excluded from the machine learning analysis.

### Survey Response and Patient-Reported Outcomes

All participants completed a baseline questionnaire, which queried their demographics and baseline health outcomes, on the Saturday following enrollment. During the study, the participants completed additional questionnaires, as described below, at intervals according to each questionnaire. All questionnaires for the overall study were administered via the web using the secure, web-based Research Electronic Data Capture system, including the stay-at-home period [35,36].

#### Depression

We used the Patient Health Questionnaire-9 (PHQ-9) to measure the severity of depression symptoms once every 2 weeks [37]. PHQ-9 contained 9 questions, with each answer being scored on a scale of 0 to 3. Higher scores indicated more severe depressive symptoms.

#### Global MS Symptom Burden

We used the Multiple Sclerosis Rating Scale—Revised (MSRS-R) to measure global MS symptom burden and neurological disability once every 4 weeks [38]. MSRS-R assessed 8 neurological domains (walking, upper limb function, vision, speech, swallowing, cognition, sensory, bladder, and bowel function); each domain scored as 0 to 4, with 0 indicating the absence of symptom and 4 indicating higher symptom burden and more severe disability.

#### Fatigue

We used the 5-item version of the Modified Fatigue Impact Scale (MFIS-5) to measure the impact of fatigue on cognitive, physical, and psychosocial function once every 4 weeks [39]. Each item in MFIS-5 was scored on a 5-point Likert scale from 0 (never) to 4 (almost always). Higher scores indicated more severe fatigue.

#### Sleep Quality

We used the Pittsburgh Sleep Quality Index (PSQI) to measure sleep disturbances once every 4 weeks [40]. PSQI comprised 19 individual items, with 7 component scores (each on a 0-3 scale) and 1 composite score (0 to 21, where higher scores indicate a poorer sleep quality).

For each outcome, we averaged the measures collected during the stay-at-home-period and then dichotomized the resulting outcomes using thresholds. The binary outcomes would likely have better clinical utility as they are more easily understood by patients (for self-monitoring), volunteers with limited mental health training, or even clinicians. For “Depression,” PHQ-9 scores were dichotomized as “≥5: presence of depression” and “<5: absence of depression.” For “Global MS symptom burden,” MSRS-R scores were dichotomized as “≥6.4: higher burden” and “<6.4: lower burden.” For “Fatigue,” MSIF-5 scores were dichotomized as “≥8: high fatigue” and “<8: low fatigue.” For “Sleep quality,” PSQI scores were dichotomized as “≥9: poorer sleep quality” and “<9: better sleep quality.” The thresholds for depression and sleep quality were based on previous works [37,41]. Given the lack of consensus from the literature, we calculated the median scores of the global MS symptom burden and fatigue in a larger data set of 104 people with MS, of which the 56 (53.8%) people with MS in this paper represented a subgroup (with data collection encompassing the stay-at-home period) and used the median scores as the thresholds.

### Sensor Data Collection

Each participant installed a mobile app based on the AWARE framework [42], which provided backend and network infrastructure that unobtrusively collected from smartphones the location, screen usage (ie, when the screen status changed to on or off and locked or unlocked), and call logs (for incoming, outgoing, and missed calls). Further, participants wore a Fitbit Inspire HR, which captured the number of steps, sleep status (asleep, awake, restless, or unknown), and heart rate. Calls and screen use were event-based sensor streams, whereas location, steps, sleep, and heart rate were time series sensor streams. We sampled location coordinates at 1 sample per 10 minutes, and steps, sleep, and heart rate at 1 sample per minute.

Data from AWARE were deidentified and automatically transferred over WiFi to a study server at regular intervals. Data from the Fitbit were retrieved using the Fitbit application programming interface at the end of the data collection. Participants were asked to keep their devices charged and to always carry their phone and wear Fitbit.

To protect confidentiality, we removed identifiable information (eg, names and contact information) from survey and sensor data prior to analysis. We followed the standard practice for sensor data security.

### Mediation Analysis

Mediation analysis was performed using the nondichotomized outcomes (ie, the average of the patient-reported outcomes collected during the stay-at-home-period). Process Macro in SPSS (IBM Corp) was used for mediation analysis [43].

### Data Processing and Machine Learning

**Figure 1:**
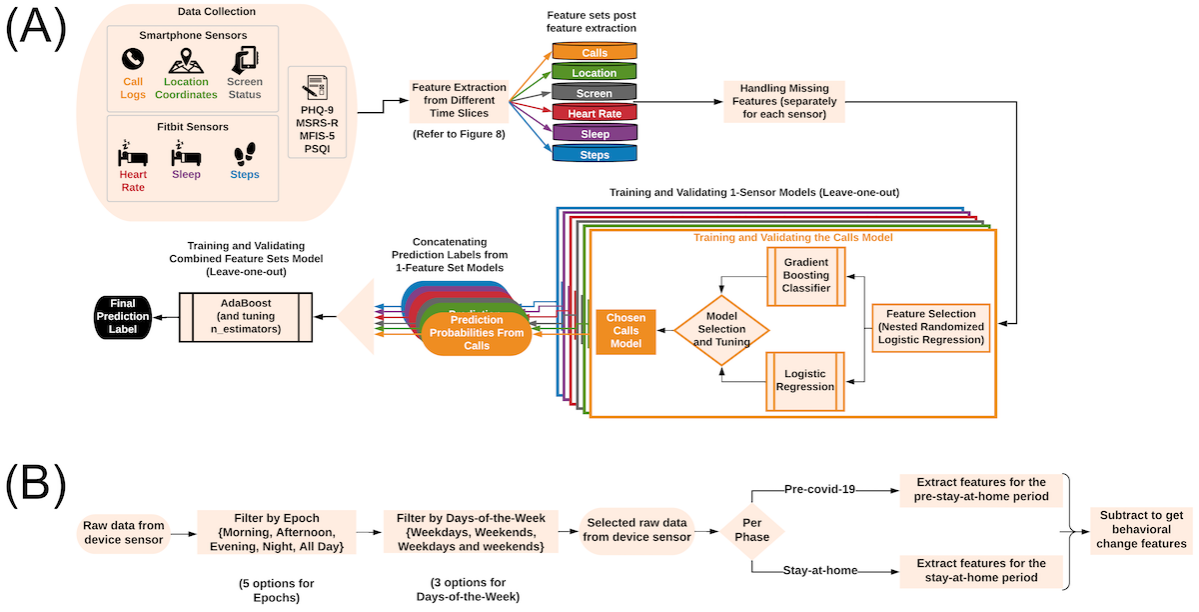
Data processing and analysis pipeline. (A) Machine learning pipeline for predicting depression (Patient Health Questionnaire-9 [PHQ-9]), global MS symptom burden (Multiple Sclerosis Rating Scale—Revised [MSRS-R]), fatigue (Modified Fatigue Impact Scale-5 [MFIS-5]), and sleep quality (Pittsburgh Sleep Quality Index [PSQI]) using passive sensors from smartphones and fitness trackers. (B) For each sensor during the pre–stay-at-home period and the stay-at-home period, each feature was extracted from 15 time slices. The pre–stay-at-home features were subtracted from the stay-at-home features to obtain the behavior change features. First, raw data from the device sensor were preprocessed and then filtered by a time-of-the-day epoch and a days-of-the-week option. Features were then extracted from the selected raw data.

The data processing and analysis pipeline (Figure 1) were built on our prior work [29] and involved several steps:

1. Feature extraction from sensors over time slices to identify behavior changes.
2. Handling missing features.
3. Machine learning to predict patient-reported health outcomes during the stay-at-home period:
  Using 1-sensor models (ie, models containing features from 1 sensor).
  Combining 1-sensor models to obtain the best model for each outcome.

#### Feature Extraction

We computed features from the 6 sensors of calls, heart rate, location, screen, sleep, and steps, given their potential to inform depressive symptoms [29,44-48], as well as fatigue [49], MS symptom burden such as decreased mobility [27], and sleep quality [50,51].

Location features captured mobility patterns. Steps and heart rate captured the extent of physical activities. Calls features captured communication patterns. Screen features might inform the ability for concentration [52,53] and the extent of sedentary behavior [54], despite potential caveats for people with MS and other chronic neurological disorders. Sleep features captured sleeping duration and patterns, which could indicate sleep disturbance (eg, insomnia or hypersomnia) associated with depression [55]. Please see Multimedia Appendix 1 (section A.1 [29,44,46,56-59]) for details of features extracted from each sensor.

Features from the 6 sensors were extracted over a range of temporal slices (Figure 1B) preceding and during the stay-at-home period. For each period, we obtained the daily averages of these features by computing the average of the daily feature values. We computed features of behavior changes by subtracting the daily averages of features during the baseline (pre–stay-at-home) period from the stay-at-home period for the machine learning models.

#### Temporal Slicing

The temporal slicing approach extracted sensor features from different time segments (Figure 1B). Past work showed that this approach can better define the relationship between a feature and depression. For example, Chow et al [60] found no relationship between depression and the time spent at home during 4-hour time windows, but they found that people with more severe depression tended to spend more time at home between 10 AM and 6 PM. Similarly, Saeb et al [61] found that the same behavioral feature calculated over weekdays and weekends could have a very different association with depression. Here, we obtained all available data (spanning multiple days of the study) from a specific epoch or time segment of the day (all day, night [ie, 12 AM-6 AM], morning [ie, 6 AM-12 PM], afternoon [ie, 12 PM-6 PM], and evening [ie, 6 PM-12 AM]) and for specific days of the week (all days of the week, weekdays only [ie, Monday-Friday], and weekends only [ie, Saturday-Sunday]) to achieve 15 data streams or temporal slices. To extract features from each of the 15 temporal slices, we first computed daily features, averaged daily features from the pre–stay-at-home period, and averaged daily features from the stay-at-home period. We then subtracted the pre–stay-at-home feature matrix from the stay-at-home feature matrix to obtain the behavior change features. We concatenated the resulting 15 temporal slices of behavior change features to derive the final feature matrix.

#### Feature Matrix

After feature extraction, each of the 6 sensors had a feature matrix, with each sample containing a participant’s feature vector comprising behavior change features from 15 different temporal slices.

#### Handling Missing Data

Missing sensor data can occasionally occur due to several reasons. Our approach for handling missing data is described in Multimedia Appendix 1 (section A.2).

#### Machine Learning Using Nested Feature Selection

We built machine learning models to predict dichotomized outcomes using the data set, building on a published approach [29], and validated our models using leave-5-participants-out cross-validation to minimize overfitting. The model generation process followed these steps:

1. Stable feature selection using randomized logistic regression, leveraging temporal slices.
2. Training and validating 1-sensor models for each of the 6 feature sets of calls, heart rate, location, screen, sleep, and steps.
3. Obtaining predictions from combinations of sensors by combining detection probabilities from 1-sensor models to identify the best performing model.
4. Classifying different outcomes
  by running the pipeline for each outcome.

#### Stable Feature Selection

To enable stable feature selection from a vast number of behavioral features, Chikersal et al [29] proposed an approach called “nested randomized logistic regression,” which we deployed in this study. This method decomposed the feature space for each sensor by grouping features from the same time slices and performed randomized logistic regression on each of these groups. The selected features from all groups (ie, all time slices) are then concatenated to give a new and much smaller set of features. Next, we performed randomized logistic regression on this new set of features to extract the final selected features for the sensor. We performed the nested feature selection for each of the six 1-sensor models, thereby nesting the process. This method was performed in a leave-5-participants-out manner such that the model used to detect an outcome for a participant did not include that person during the feature selection process. More details about this method can be found in Multimedia Appendix 1 (section A.3).

#### Training and Validating 1-Sensor Models

For each sensor, we built a model of the selected features from that sensor to detect an outcome. We used leave-5-participants-out cross-validation to choose the parameters for that model. We trained models using the following 2 machine learning algorithms: logistic regression and gradient boosting classifier [29]. We chose the model with the best F_1_-score for a given outcome, which provides the detection probabilities for the outcome. The process is independent of other outcomes.

#### Obtaining Predictions From Combinations of Sensors

The detection probabilities from all six 1-sensor models were concatenated into a single feature vector and given as input to an ensemble classifier (ie, AdaBoost with gradient boosting classifier as a base estimator), which then outputted the final label for the outcome. For all outcomes, only the detection probabilities of the positive label “1” were concatenated. The positive label was the “presence of depression” for “depression,” “high burden” for “global MS symptom burden,” “severe fatigue” for “fatigue,” and “poor sleep quality” for “sleep quality.” The “n_estimators” (the maximum number of estimators at which boosting is terminated) parameter was tuned during leave-5-participants-out cross-validation to achieve the best-performing combined model.

To analyze the usefulness of each sensor, we implemented a feature ablation analysis by generating detection results for all possible combinations of 1-sensor models. For six 1-sensor models, there were 57 combinations of feature sets, as the total combinations = combinations with 2 sensors + ... + combinations with 6 sensors = 



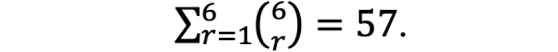



#### Classifying Different Outcomes

This pipeline of training and validating six 1-sensor models and 57 combined models was run independently for each of the 4 outcomes. For each outcome, we reported the performance based on the best combination of sensors. We also reported the performance of baseline models (ie, a simple majority classifier whereby every point is assigned to whichever is in the majority in the training set) as well as models containing all 6 sensors.

## Results

### Participant Characteristics

The characteristics of the 56 participants were representative of the typical MS study (median age 43.5 years; n=48, 86% women). Table 1 shows the detailed participant characteristics.

**Table 1:** Study participant characteristics.

Variable		Value
**Sex, n (%)**		
	Female	48 (86)
	Male	8 (14)
**Race, n (%)**		
	White	51 (91)
	African or African American	5 (9)
**Ethnicity, n (%)**		
	Non-Hispanic or Latino	55 (98)
	Hispanic or Latino	1 (2)
Age (years), median (IQR)		43.5 (37-52)
Time elapsed (years) from age of first neurological symptom onset to study participation, median (IQR)		13.0 (6.7-17.4)
PDDS^a^ score at start of study, median (IQR)		1 (0-3)
**Disease-modifying treatment, n (%)**		
	Higher efficacy	38 (68)
	Standard efficacy	12 (21)
**Depression diagnosis, n (%)**		
	Not diagnosed with clinical depression before study enrollment	39 (70)
	Diagnosed with clinical depression before study enrollment	17 (30)
**Pharmacotherapy for depression, n (%)**		
	Not taking medication for depression before study enrollment	39 (70)
	Taking medication for depression before study enrollment	17 (30)
**Nonpharmacotherapy for depression, n (%)**		
	Not receiving nonmedication therapy for depression before study enrollment	52 (93)
	Receiving nonmedication therapy for depression before study enrollment	4 (7)
**Study outcomes: average measures during the stay-at-home period, median (IQR)**		
	PHQ-9^b^ (depression)	3.7 (0.0-7.4)
	MSRS-R^c^ (global MS^d^ symptom burden)	7.5 (3.4-10.3)
	MFIS-5^e^ (fatigue)	8.0 (4.6-11.0)
	PSQI^f^ (sleep quality)	11.0 (7.8-14.3)

^a^PDDS: Patient Determined Disease Steps.

^b^PHQ-9: Patient Health Questionnaire-9.

^c^MSRS-R: Multiple Sclerosis Rating Scale—Revised.

^d^MS: multiple sclerosis.

^e^MFIS-5: Modified Fatigue Impact Scale-5.

^f^PSQI: Pittsburgh Sleep Quality Index.

### Interrelated Outcomes

**Figure 2:**
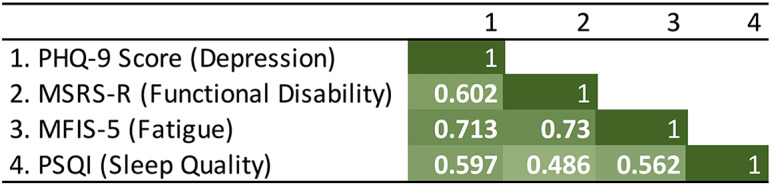
Correlations among the 4 clinically relevant patient-reported outcomes in this study. For all correlations, *P*<.001 (N=56). MFIS-5: Modified Fatigue Impact Scale-5; MSRS-R: Multiple Sclerosis Rating Scale—Revised; PHQ-9: Patient Health Questionnaire-9; PSQI: Pittsburgh Sleep Quality Index.

The main study outcome is patient-reported depression as well as associated neurological symptom burden, fatigue, and sleep quality. We measured the Pearson correlations among the average values of the 4 outcomes during the stay-at-home period for the participants. Depression severity (PHQ-9) correlated with the global MS symptom burden (MSRS-R), fatigue severity (MFIS-5), and sleep quality (PSQI; Figure 2).

**Figure 3:**
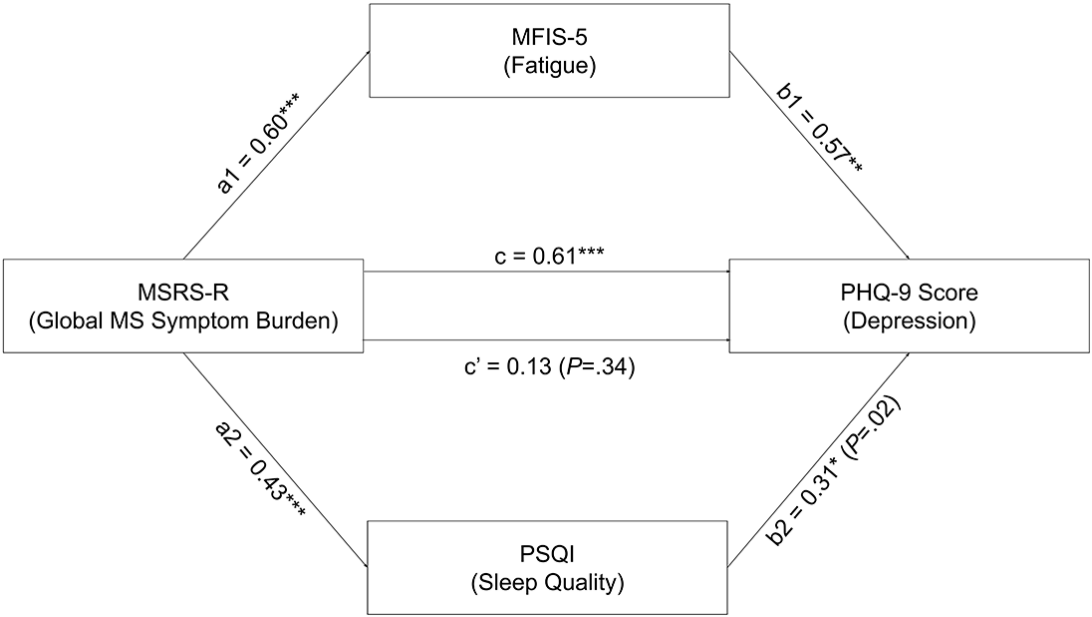
Parallel mediation analysis. Path model showing the effect of Multiple Sclerosis Rating Scale—Revised (MSRS-R; measuring global MS symptom burden) on the Patient Health Questionnaire-9 (PHQ-9) score (measuring depression) as mediated simultaneously by Modified Fatigue Impact Scale-5 (MFIS-5; measuring fatigue) and Pittsburgh Sleep Quality Index (PSQI; measuring sleep quality). Path c represents the effect of MSRS-R on PHQ-9 without mediators in the model. Path c’ represents the effect of MSRS-R on PHQ-9 when MFIS-5 and PSQI mediators are included in the model. Paths a1b1 and a2b2 represent the effect of MSRS-R on PHQ-9 through MFIS-5 or PSQI respectively. The figure shows nonstandardized β regression coefficients (*P<.05, **P<.001, ***P<.0001) as reported by PROCESS Macro in SPSS [43]. MS: multiple sclerosis.

To dissect the complex relationship among these outcomes to inform better patient monitoring and guide potentially more precise interventions, we performed mediation analysis (Figure 3). When MFIS-5 and PSQI were both included as mediators in the model (path c’), the association between MSRS-R and PHQ-9 was no longer significant (effect size=0.13 and the bias-corrected bootstrap confidence intervals=–0.14 and 0.40). However, the association between MSRS-R and PHQ-9 through MFIS-5 (path a1b1) remained significant (effect size=0.34 and the bias-corrected bootstrap confidence intervals=0.13-0.52). The association between MSRS-R and PHQ-9 through PSQI (path a2b2) also remained significant (effect size=0.13 and the bias-corrected bootstrap confidence intervals=0.02-0.27). Hence, the relationship between the global MS symptom burden and depression might be mediated by both fatigue and sleep quality.

### Predicting Outcomes During the Stay-at-home Period

**Figure 4:**
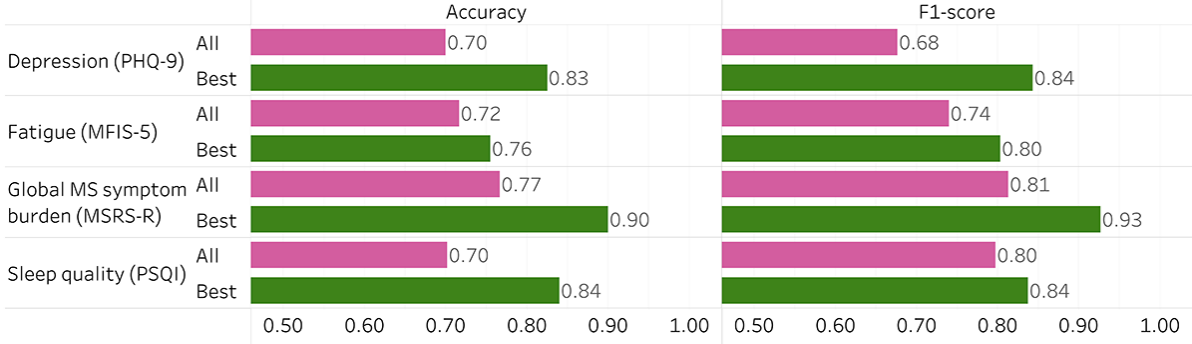
Performance of the machine learning pipeline using all sensors and the best sensor combination for predicting each of the 4 clinically relevant outcomes in people with multiple sclerosis during a state-mandated stay-at-home period. "Accuracy (All Sensors)" and "F1 Score (All Sensors)" are the accuracy (× 0.01) and F1-score obtained by combining all 6 sensors. "Accuracy (Best Sensors)" and "F1 Score (Best Sensors)" are the accuracy (× 0.01) and F1-score obtained by the best combination of sensors. See Multimedia Appendix 1 for additional performance metrics of all models. MFIS-5: Modified Fatigue Impact Scale-5; MSR-R: Multiple Sclerosis Rating Scale—Revised; PHQ-9: Patient Health Questionnaire-9; PSQI: Pittsburgh Sleep Quality Index.

Figure 4 shows the performance of the machine learning pipeline for predicting each of the 4 outcomes using the best sensor combinations (ie, the set of sensors that had the best performance for each outcome). Accuracy is the percentage of patients for whom the outcome label was correctly predicted. F_1_-score is a metric of model performance that measures the harmonic mean of precision and recall. Precision is the positive predictive value, or the number of true positive labels divided by the number of all positive labels (true positive + false positive). Recall is sensitivity, or the number of true positive labels divided by the number of all patients who should have the positive labels (true positive + false negative). In this study, “positive” label refers to the outcome of interest (eg, presence of depression is the positive label for depression). Figures S1 to S4 in Multimedia Appendix 1 report the performance of individual sensors and when all 6 sensors were included. Tables S1 to S4 in Multimedia Appendix 1 list the features selected by the best models for each outcome, and their corresponding coefficients.

#### Depression

The baseline model (simple majority classifier) had an accuracy of 50.0% in predicting the presence of depression during the stay-at-home period. The model containing all sensors had an accuracy of 70% (40% improvement over the baseline). The model with the best combination of sensors (calls, heart rate, and location) had an accuracy of 82.5% (65% improvement over the baseline).

#### Global MS Symptom Burden

The baseline model had an accuracy of 64.7% in predicting high global MS symptom burden (versus “low burden”) during the stay-at-home period. The model containing all sensors had an accuracy of 76.7% (18.5% improvement over the baseline). The model with the best combination of sensors (calls, heart rate, location, and screen) had an accuracy of 90% (39% improvement over the baseline).

#### Fatigue

The baseline model had an accuracy of 61.8% in predicting severe fatigue (versus “mild fatigue”) during the stay-at-home period. The model containing all sensors had an accuracy of 71.7% (16% improvement over the baseline). The model with the best combination of sensors (calls, heart rate, and location) had an accuracy of 75.5% (22% improvement over the baseline)

#### Sleep Quality

The baseline model had an accuracy of 65.7% in predicting poor sleep quality (ie, “poor sleep quality” versus “better sleep quality”) during the stay-at-home period. The model containing all sensors had an accuracy of 70.2% (7% improvement over the baseline). The model with the best combination of sensors (location and screen) had an accuracy of 84% (28% improvement over the baseline).

## Discussion

### Principal Findings

In this unique natural experiment conducted during the early wave of the COVID-19 pandemic, we reported the clinical utility of digital phenotyping for predicting clinically relevant outcomes for people with MS. Using only passively sensed data, our machine-learning models predicted the presence of depression, high global MS symptom burden, severe fatigue, and poor sleep quality during the stay-at-home period with potentially clinically actionable performance.

The best models outperformed not only baseline models (simple majority classifier) but also models containing all sensors. The best sensor combinations for predicting depression and fatigue were the same (ie, calls, heart rate, and location), while these sensors were also included in the best sensor combination for predicting global MS symptom burden (ie, calls, heart rate, location, and screen). Comparably, the best sensor combination for sleep quality (ie, location and screen) had the smallest overlap with the sensor combinations for the other three outcomes. This observation was consistent with the finding that depression, fatigue, and global MS symptom burden were better correlated among themselves than with sleep quality (Figure 2). We also looked at the feature coefficients of the features selected by the best models (Multimedia Appendix 1, section B.2). Examples of the best features of changed behavior selected by the best model for predicting depression (ie, features with the highest absolute coefficients) include increase in number of incoming calls during evenings on weekdays, decrease in average heart rate when the person is at rest or has low activity (outside exercise heart rate zones) during evenings on weekends, and increase in regularity in movement patterns in 24-hour periods with respect to nights on weekends.

Our findings built on a small body of prior work that explored the feasibility of passive sensing in people with MS and preliminary correlations between passively sensed behaviors and MS outcomes. For example, Newland et al [26] explored real-time depth sensors at home to identify gait disturbance and falls in 21 patients with MS. Other studies reported correlations between passively sensed physical activity and disability worsening in people with MS [27,62,63]. Chitnis et al [28] examined the gait, mobility, and sleep of 25 people with MS over 8 weeks using sensors mounted on their wrist, ankle, and sternum, and reported correlations among gait-related features (eg, turn angle and maximum angular velocity), sleep and activity, and disability outcomes.

Previous work on predicting health outcomes for people with MS using passively sensed behaviors is scarce. Tong et al [49] used passively sensed sleep and activity data collected from 198 people with MS over 6 months to predict fatigue severity and overall health scores, achieving good performance in line with acceptable instrument errors. To our knowledge, our study is the first to use passively sensed behavior changes to predict multiple interrelated clinically relevant health outcomes in MS, including depression, disability, fatigue, and sleep quality. While several studies used passively sensed data from the general population to report behavior changes during the COVID-19 pandemic [64-67], our study provides the first real-world evidence of potential clinical utility of passively sensed behavior changes to predict health outcomes during the unique stay-at-home period in a population with a chronic neurological disorder and complex health needs. From a methodological standpoint, the application of behavioral features computed over temporal slices to predict depression and other health outcomes in people with MS is novel. Our approach of using change in features between the period preceding the stay-at-home and stay-at-home periods to predict outcomes during the stay-at-home period is also novel. Finally, we included new heart rate features that can be computed using data from the Fitbit application programming interface.

Our approach has potential clinical utility, particularly during major stressful events (beyond COVID-19) that worsen health outcomes and limit health care access. For instance, predictive models built using our approach could help patients self-monitor their health when access to in-person clinical care becomes suddenly limited and could encourage patients (or their caregivers) to actively seek medical attention sooner when the models predict adverse outcomes. Further, our models could help clinicians better monitor at-risk patients and make triage decisions for patients who require prioritization for interventions (eg, medication and counseling), particularly in the setting of suddenly limited health care access and scarce resources.

### Limitations

Our study has 2 limitations. First, the COVID-19 pandemic started during our data collection for an ongoing larger study of people with MS. While it provided a unique opportunity to conduct a natural experiment to assess the utility of digital phenotyping to predict health outcomes in people with MS during the highly unusual stay-at-home period, we had a modest sample size of participants who happened to have sufficient sensor data collected both just before the sudden issue of the stay-at-home order and during the stay-at-home period. We also had limited ability to seek external replication of the drastic behavior changes during the early stage of the pandemic since the stay-at-home order was lifted and has not been reinstated. To reduce the chance of overfitting and improve the validity of the findings, we used leave-5-participants-out cross-validation, such that in each fold, the participants used for training and testing were different. Our approach performed well for not only 1 outcome but all 4 clinically relevant outcomes pertaining to mental health and neurological disability in people with MS. We have reasonable confidence because of the consistently good model performance across all 5 folds and the consistently robust predictions for all 4 outcomes. We are not aware of other published studies with data from before and during the stay-at-home orders, particularly involving patient population with chronic neurological disorders such as MS who are at heightened risk for adverse health outcomes resulting from social isolation, reduced support, and limited health care access. Given the uniqueness of the data set, we believe the findings are clinically relevant despite the relatively modest sample size. Second, the study used patient-reported health outcomes. Given the restriction of in-person clinical visits during the stay-at-home period, rater-performed examination was not feasible. Importantly, these patient-reported outcomes are all validated for people with MS, highly correlated with rater-determined measures, interrelated among themselves, and clinically relevant.

In summary, we reported the potential clinical utility of digital phenotyping in predicting subsequent health outcomes in people with MS during a COVID-19 stay-at-home period. Specifically, we predicted the presence of depression, high global MS symptom burden, severe fatigue, and poor sleep quality in people with MS during the stay-at-home period using passively sensed behavior changes measured by smartphone and wearable fitness tracker. The predictive models achieved potentially clinically actionable performance for all 4 outcomes. This study paved the way for future replication studies during major stressful events and has implications for future patient self-monitoring and clinician screening for urgent interventions in MS and other complex chronic diseases.

## Appendix

Multimedia Appendix 1 Supplementary material.

## Abbreviations

MFIS-5: Modified Fatigue Impact Scale-5
MS: multiple sclerosis
MSRS-R: Multiple Sclerosis Rating Scale—Revised
PHQ-9: Patient Health Questionnaire-9
PSQI: Pittsburgh Sleep Quality Index
