# Supplementary material for: Predicting Multiple Sclerosis Outcomes During the COVID-19 Stay-at-home Period: Observational Study Using Passively Sensed Behaviors and Digital Phenotyping
Source: JMIR Ment Health. 2022 Aug 24;9(8):e38495. doi: 10.2196/38495 (PMC9407162; doi:10.2196/38495)
Supplement: Multimedia Appendix 1 [file mental_v9i8e38495_app1.docx]

## Appendix

### A. Supplementary Methods

#### A.1. Feature Extraction

*Calls Features:* Calls features were calculated using the call logs from the smartphone. We extracted the following features: *Number and duration of all incoming, outgoing, and missed calls, number of correspondents overall*.

*Heart Rate Features:* Heart rate features were calculated from the heart rate over time using the Fitbit API. The following features were calculated: *Mean heart rate, total time spent in the “fat burn” heart rate zone, total time spent in the “cardio” heart rate zone, total time spent in the “peak” heart rate zone, and total time spent below any heart rate zones indicating exercise (i.e., the out of the range zone).* The fat burn, cardio, and peak heart rate zones were calculated by Fitbit for each person separately.

*Location Features:* Location features were derived from the Location ‘virtual’ sensor of the smartphone which provided the best estimate of location based on available GPS, WiFi and cellular tower signals. We extracted the following Location features: *Location variance* (sum of the variance in latitude and longitude coordinates), *log of location variance*, and *total distance traveled*. *Circadian movement* [44] was calculated using the Lomb-Scargle method [56]. It encoded the extent to which a person’s location patterns followed a 24-hour circadian cycle.

Next, we labeled location samples as “static” or “moving” and clustered the “static” samples as described by Chikersal et. al.[29]. We then extracted: *number of significant places, radius of gyration* [46]*, percentage of time spent at top-3 (most frequented) places, percentage of time spent moving, and percentage of time spent in insignificant or rarely visited locations*. We further calculated the *average and standard deviation of length of stay at significant places* as well as *Location entropy* and *normalized location entropy across significant places (*using previously described method [44]). Higher location entropy occurred when time is spent evenly across significant places.

We assumed the place most visited by the participant at late night (between 00:00 hrs to 06:00 hrs) to be their home location and the place most visited by the participant during the afternoon (between 12:00 hrs to 18:00 hrs) to be their work location. To compute home location, we clustered the location coordinates from all nights and assumed the center of the most frequented cluster to be the participant’s home location center. To compute work location, we clustered the location coordinates from all afternoons and assumed the center of the most frequented cluster to be the participant’s work location center. We used the self-reported home addresses of the participant to verify their home location, and switch home and work locations if the computed home location was found to not match with their self-reported home addresses. We calculated the *time spent at home, assuming home to be within 100 meters of the home location* *center* based on the default geofencing radius used by automation systems like HomeKit and <https://www.home-assistant.io/>.

*Screen Features:* Screen features were calculated using the screen status sensor in the smartphone, which recorded screen status (on, off, lock, unlock) over time. We extracted the following phone usage features: *total number of unlocks, mean number of unlocks per minute, total time spent interacting with the phone, and the median length of bouts (or continuous periods of time) during which the participant was interacting with the phone and when the screen was unlocked*. A participant was noted to be “interacting” with the phone during the interval between "unlocked" (on) and "locked" (off) screen status.

*Sleep Features:* Sleep features were calculated from the daily sleep summaries and minute-to-minute sleep inferences (asleep, restless, awake, unknown) using the Fitbit API. Sleep captured by Fitbit is accurate +/- 45 minutes [57-59]. The following features were calculated from all sleep data: *total minutes asleep, total time in bed, and total sleep records*. The following features were calculated from the sleep data for the longest sleep record or “main” sleep period after excluding any short naps: *total time spent sleeping during main sleep, total time spent in bed during main sleep, sleep efficiency during main sleep,* which was calculated as (time asleep / (total time in bed - time to fall asleep))*, total time spent restless during main sleep, total number of times restless during main sleep, start time of main sleep in hours from midnight, and end time of main sleep in hours from midnight*.

*Steps Features:* Steps features were calculated from the step counts over time using the Fitbit API. The following features were calculated: *total number of steps, the number of minutes labeled as “sedentary” by Fitbit, the number of minutes labeled as “lightly active” by Fitbit, the number of minutes labeled as “fairly active” by Fitbit, and the number of minutes labeled as “very active” by Fitbit*.

#### A.2. Handing Missing Data

Missing sensor data can occasionally occur due to technical issues (e.g., non-functioning phone/app/server, faulty or delayed data transfer) or compliance issues (e.g., participant not carrying the smartphone or wearing the FitBit) but more often due to semantic reasons. For example, if a participant made and received 0 calls during a period, there would be no calls data. Thus, we encoded missing data into features since we could not differentiate whether such data were not collected or did not exist to be collected due to semantic reasons.

A missing feature during a time slice for many participants could indicate non-semantic issues such as non-functioning server. Further, a participant with many missing features could indicate non-semantic issues such as the non-functioning phone/app. To empirically determine the thresholds, we plotted the number of participants and features remaining for various thresholds and noted the largest differential in curves. Hence, we excluded all features (in a time slice) with missing values in more than 14 participants and likewise excluded participants missing more than 20% of all features. For each feature we calculated the minimum feature value, and imputed missing features as that value minus 1. As we handled missing data independently across feature time slices, the number of participants and features were different across sensors as missing features in each feature set.

#### A.3. Stable Feature Selection Formulation

Best(Fs) = sel(concatenate[sel(Fs1),sel(Fs2),...,sel(FsT )]) where Fsi = features from sensor s and time slice i (e.g. calls features from the mornings on weekdays calculated weekly), T = total number of time slices, and sel(...) is the Randomized Logistic Regression Function. T = 15. Best(Fs) are the final features selected from sensor s and are given as input to the 1-sensor model for sensor s.

### B. Supplementary Results

#### B.1. Prediction Results

Figure S1 shows the performance measures for models built when using behavioral change features from each sensor separately, the combination of all sensors (*i.e.*, “All 6”), and the best sensor combination (*i.e.*, “Calls, Heart rate, Location”) for detecting the presence of depression (*i.e.*, “depression” vs. “no depression”) during the stay-at-home period. The best sensor combination is the sensor combination that gives us the best f1-score out of the 63 different combinations of the 6 sensors tried during a feature ablation study. The number of participants may be different for models containing different sensors due to missing data (see methods section).

*Figure S1. Model performance (accuracy, f1-score, precision, and recall) for predicting depression in pwMS during the state mandated stay-at-home period.

*

Figure S2 shows the performance for predicting the global MS symptom burden (*i.e.,*, “high disability” vs. “low disability”) during the stay-at-home period for the different models.

Figure S3 shows the performance for predicting fatigue (*i.e.*, “severe fatigue” vs. “modest fatigue”) during the stay-at-home period for the different models.

Figure S4 shows the performance for predicting sleep quality (*i.e.*, “poor sleep quality” vs. “better sleep quality”) during the stay-at-home period for the different models.

*Figure S2. Model performance (accuracy, f1-score, precision, and recall) for predicting global MS symptom burden in pwMS during the state mandated stay-at-home period.

*

*Figure S3. Model performance (accuracy, f1-score, precision, and recall) for predicting fatigue in pwMS during the state mandated COVID-19 stay-at-home period.*

*

*

*Figure S4. Model performance (accuracy, f1-score, precision, and recall) for predicting sleep quality in pwMS during the state mandated COVID-19 stay-at-home period.

*

#### B.2. Coefficients of Features Selected by the Best Models

For each outcome, the tables below list the features selected by the best 1-sensor models along with the number of folds with which they were selected and the mean coefficient of those features across all folds in which they were selected. The feature values indicate the change in the features between the pre-stay-at-home period and the stay-at-home period (*i.e.,* the feature value during the stay-at-home period minus the feature value before the stay-at-home period). The higher the *absolute* mean coefficient across folds indicates the greater importance of the feature.

Table S1 shows the features selected by the best 1-sensor models used to predict the presence of depression. The best model for depression contains calls, heart rate and location. Examples of the best features from these sensors (*i.e.*, features with the highest absolute coefficients) include the change in number of incoming calls during evenings on weekdays, change in average heart rate when person is at rest or has low activity during evenings on weekends, and regularity in movement patterns in 24-hour periods with respect to nights of weekends.

Table S1. Features selected by the best 1-sensor models when predicting has depression vs. no depression. SNO: Serial number.

| **SNO** | **Sensor** | **Feature** | **Time-of-the-Day** | **Days-of-the-Week** | **Number of Folds Selected** | **Mean Coefficient Across Folds** |
| --- | --- | --- | --- | --- | --- | --- |
| 1 | Calls | Number of incoming calls | Evening | Weekday | 1 | 0.52 |
| 2 | Calls | Duration of incoming calls | Morning | Weekday | 1 | 0.4 |
| 3 | Calls | Duration of incoming calls | Afternoon | Weekday | 5 | 0.34 |
| 4 | Calls | Duration of incoming calls | Afternoon | All | 1 | 0.31 |
| 5 | Calls | Duration of incoming calls | Evening | Weekday | 2 | 0.31 |
| 6 | Calls | Duration of incoming calls | Evening | All | 3 | 0.28 |
| 7 | Calls | Number of outgoing calls | Afternoon | Weekend | 1 | 0.12 |
| 8 | Calls | Duration of incoming calls | Evening | Weekend | 2 | 0.12 |
| 9 | Calls | Number of correspondents | All | Weekend | 1 | 0.08 |
| 10 | Heart Rate | Average heart rate outside exercise heart rate zones (i.e., when person is at rest or has low activity) | Night | Weekend | 1 | -1.05 |
| 11 | Heart Rate | Average heart rate outside exercise heart rate zones (i.e., when person is at rest or has low activity) | Evening | Weekend | 3 | -0.91 |
| 12 | Heart Rate | Time spent in the fat burn heart rate zone | Morning | Weekend | 4 | 0.69 |
| 13 | Heart Rate | Time spent in peak heart rate zone | Evening | Weekend | 1 | -0.63 |
| 14 | Heart Rate | Time spent outside exercise heart rate zones (i.e., when person is at rest or has low activity) | Morning | All | 1 | -0.59 |
| 15 | Heart Rate | Time spent in peak heart rate zone | Night | Weekend | 1 | -0.36 |
| 16 | Heart Rate | Time spent outside exercise heart rate zones (i.e., when person is at rest or has low activity) | Morning | Weekend | 2 | -0.27 |
| 17 | Heart Rate | Time spent in peak heart rate zone | Afternoon | Weekend | 1 | 0.14 |
| 18 | Location | Percentage of time spent at home | Morning | Weekday | 2 | -0.72 |
| 19 | Location | Circadian movement i.e., regularity in movement patterns in 24 hr periods | Morning | Weekend | 1 | 0.64 |
| 20 | Location | Location variance | Evening | Weekend | 1 | 0.62 |
| 21 | Location | Circadian movement i.e., regularity in movement patterns in 24 hr periods | Night | Weekend | 4 | 0.6 |
| 22 | Location | Percentage of time spent at the most frequented (primary) location | Evening | Weekday | 1 | 0.58 |
| 23 | Location | Standard deviation of the duration of stay at most frequented (significant) locations | Night | Weekend | 1 | 0.56 |
| 24 | Location | Standard deviation of the duration of stay at most frequented (significant) locations | All | Weekday | 5 | -0.55 |
| 25 | Location | Standard deviation of the duration of stay at most frequented (significant) locations | All | All | 2 | -0.54 |
| 26 | Location | Location entropy i.e., evenness in time spent across most frequented (significant) locations | Evening | Weekend | 1 | 0.53 |
| 27 | Location | Circadian movement i.e., regularity in movement patterns in 24 hr periods | Morning | Weekday | 1 | 0.52 |
| 28 | Location | Percentage of time spent at rarely visited locations (e.g. new places) | Afternoon | All | 4 | -0.51 |
| 29 | Location | Number of most frequented (significant) locations | All | Weekend | 1 | 0.48 |
| 30 | Location | Average duration of stay at most frequented (significant) locations | Evening | Weekend | 1 | 0.47 |
| 31 | Location | Standard deviation of the duration of stay at most frequented (significant) locations | Afternoon | Weekend | 2 | 0.42 |
| 32 | Location | Standard deviation of the duration of stay at most frequented (significant) locations | Afternoon | All | 1 | 0.42 |
| 33 | Location | Average duration of stay at most frequented (significant) locations | Morning | Weekday | 1 | -0.41 |
| 34 | Location | Location variance | Afternoon | Weekday | 1 | -0.4 |
| 35 | Location | Average duration of stay at most frequented (significant) locations | Night | Weekend | 1 | -0.38 |
| 36 | Location | Number of most frequented (significant) locations | Night | Weekend | 1 | 0.37 |
| 37 | Location | Percentage of time spent at the third most frequented location | Evening | All | 1 | -0.35 |
| 38 | Location | Percentage of time spent at home | Morning | All | 2 | -0.35 |
| 39 | Location | Circadian movement i.e., regularity in movement patterns in 24 hr periods | Afternoon | Weekday | 1 | 0.35 |
| 40 | Location | Location entropy i.e., evenness in time spent across most frequented (significant) locations | Morning | Weekday | 1 | 0.34 |
| 41 | Location | Percentage of time spent at home | Night | All | 3 | -0.33 |
| 42 | Location | Standard deviation of the duration of stay at most frequented (significant) locations | Morning | Weekend | 3 | -0.33 |
| 43 | Location | Percentage of time spent at rarely visited locations (e.g. new places) | Evening | All | 2 | -0.31 |
| 44 | Location | Percentage of time spent in motion (e.g. while driving, in public transit) | Morning | All | 1 | 0.29 |
| 45 | Location | Percentage of time spent at home | Evening | Weekday | 1 | 0.28 |
| 46 | Location | Location variance | All | Weekday | 1 | -0.28 |
| 47 | Location | Logarithm of location variance | Evening | Weekend | 3 | 0.27 |
| 48 | Location | Standard deviation of the duration of stay at most frequented (significant) locations | Evening | Weekend | 1 | 0.26 |
| 49 | Location | Average duration of stay at most frequented (significant) locations | Evening | Weekday | 2 | 0.22 |
| 50 | Location | Radius of the area covered during mobility | Afternoon | Weekday | 1 | -0.19 |
| 51 | Location | Average duration of stay at most frequented (significant) locations | Morning | All | 1 | -0.12 |
| 52 | Location | Percentage of time spent at rarely visited locations (e.g. new places) | Afternoon | Weekday | 2 | -0.1 |
| 53 | Location | Percentage of time spent at the third most frequented location | Afternoon | Weekday | 1 | 0.09 |
| 54 | Screen | Number of unlocks | Morning | Weekend | 4 | 0.12 |
| 55 | Screen | Number of unlocks | Night | Weekend | 1 | 0.12 |
| 56 | Screen | Total time spent interacting with the phone | Afternoon | Weekend | 3 | 0.12 |
| 57 | Screen | Total time spent interacting with the phone | Night | Weekday | 2 | 0.12 |
| 58 | Screen | Number of unlocks | Afternoon | Weekday | 2 | 0.11 |
| 59 | Screen | Total time spent interacting with the phone | All | All | 3 | 0.11 |
| 60 | Screen | Total time spent interacting with the phone | Night | All | 1 | 0.09 |
| 61 | Screen | Number of unlocks | Night | All | 2 | 0.08 |
| 62 | Screen | Number of unlocks | All | Weekend | 3 | 0.08 |
| 63 | Screen | Median time spent interacting with the phone per interaction or use | Night | All | 2 | 0.07 |
| 64 | Screen | Mean unlocks per minute | Night | All | 5 | 0.07 |
| 65 | Screen | Total time spent interacting with the phone | All | Weekday | 5 | 0.06 |
| 66 | Screen | Number of unlocks | All | Weekday | 2 | 0.05 |
| 67 | Screen | Number of unlocks | Evening | Weekday | 1 | 0.05 |
| 68 | Screen | Total time spent interacting with the phone | Evening | All | 3 | 0.05 |
| 69 | Screen | Median time spent interacting with the phone per interaction or use | Evening | All | 2 | 0.05 |
| 70 | Screen | Number of unlocks | Night | Weekday | 1 | 0.05 |
| 71 | Screen | Mean unlocks per minute | Night | Weekday | 5 | 0.05 |
| 72 | Screen | Median time spent interacting with the phone per interaction or use | Afternoon | All | 1 | 0.04 |
| 73 | Screen | Total time spent interacting with the phone | Evening | Weekday | 4 | 0.04 |
| 74 | Screen | Mean unlocks per minute | All | Weekend | 2 | 0.04 |
| 75 | Screen | Number of unlocks | Afternoon | Weekend | 4 | 0.04 |
| 76 | Screen | Median time spent interacting with the phone per interaction or use | Night | Weekend | 2 | 0.03 |
| 77 | Screen | Total time spent interacting with the phone | Afternoon | All | 5 | 0.03 |
| 78 | Screen | Number of unlocks | Evening | All | 1 | 0.03 |
| 79 | Screen | Median time spent interacting with the phone per interaction or use | Morning | Weekend | 4 | 0.03 |
| 80 | Screen | Mean unlocks per minute | Afternoon | Weekday | 2 | 0.03 |
| 81 | Screen | Mean unlocks per minute | Morning | Weekday | 2 | 0.03 |
| 82 | Screen | Total time spent interacting with the phone | Morning | Weekday | 4 | 0.02 |
| 83 | Screen | Median time spent interacting with the phone per interaction or use | Morning | All | 2 | 0.02 |
| 84 | Screen | Total time spent interacting with the phone | Morning | Weekend | 2 | 0.02 |
| 85 | Screen | Mean unlocks per minute | Evening | Weekend | 1 | 0.02 |
| 86 | Screen | Number of unlocks | Morning | All | 1 | 0.01 |
| 87 | Screen | Mean unlocks per minute | Afternoon | Weekend | 3 | 0.01 |
| 88 | Screen | Median time spent interacting with the phone per interaction or use | Afternoon | Weekend | 3 | 0.01 |
| 89 | Screen | Median time spent interacting with the phone per interaction or use | Night | Weekday | 1 | 0.01 |
| 90 | Screen | Median time spent interacting with the phone per interaction or use | All | Weekend | 1 | 0.01 |
| 91 | Screen | Total time spent interacting with the phone | Night | Weekend | 2 | 0.01 |
| 92 | Screen | Mean unlocks per minute | Night | Weekend | 1 | 0.01 |
| 93 | Screen | Number of unlocks | Evening | Weekend | 1 | 0 |
| 94 | Screen | Median time spent interacting with the phone per interaction or use | Evening | Weekday | 1 | 0 |
| 95 | Sleep | Total sleep records/ bouts | Evening | All | 4 | 0.19 |
| 96 | Sleep | Total times restless during the main sleep (i.e., the longest sleep bout) | All | Weekend | 4 | 0.18 |
| 97 | Sleep | Total time between midnight and the start time of the main sleep (i.e., the longest sleep bout) | Night | All | 1 | 0.17 |
| 98 | Sleep | Total duration restless during the main sleep (i.e., the longest sleep bout) | All | Weekend | 1 | 0.16 |
| 99 | Sleep | Total sleep records/ bouts | Morning | Weekday | 5 | 0.14 |
| 100 | Sleep | Total time spent asleep | All | Weekend | 1 | 0.12 |
| 101 | Sleep | Total sleep records/ bouts | All | Weekday | 2 | 0.11 |
| 102 | Sleep | Time spent asleep during the main sleep (i.e., the longest sleep bout) | Morning | Weekend | 1 | 0.11 |
| 103 | Sleep | Total sleep records/ bouts | Night | All | 3 | 0.1 |
| 104 | Sleep | Total times restless during the main sleep (i.e., the longest sleep bout) | Morning | Weekend | 1 | 0.09 |
| 105 | Sleep | Total times restless during the main sleep (i.e., the longest sleep bout) | Morning | All | 1 | 0.09 |
| 106 | Sleep | Total time spent asleep | Evening | Weekday | 2 | 0.08 |
| 107 | Sleep | Total times restless during the main sleep (i.e., the longest sleep bout) | Night | Weekend | 1 | 0.07 |
| 108 | Sleep | Total time between midnight and the end time of the main sleep (i.e., the longest sleep bout) | All | Weekend | 1 | 0.07 |
| 109 | Sleep | Total duration restless during the main sleep (i.e., the longest sleep bout) | Morning | All | 4 | 0.07 |
| 110 | Sleep | Total duration restless during the main sleep (i.e., the longest sleep bout) | Morning | Weekend | 2 | 0.07 |
| 111 | Sleep | Total time between midnight and the end time of the main sleep (i.e., the longest sleep bout) | Evening | All | 1 | 0.07 |
| 112 | Sleep | Total time spent asleep | Morning | All | 1 | 0.06 |
| 113 | Sleep | Total duration restless during the main sleep (i.e., the longest sleep bout) | Evening | Weekday | 2 | 0.06 |
| 114 | Sleep | Total time between midnight and the start time of the main sleep (i.e., the longest sleep bout) | Morning | Weekday | 1 | 0.06 |
| 115 | Sleep | Total sleep records/ bouts | All | Weekend | 1 | 0.04 |
| 116 | Sleep | Total time between midnight and the end time of the main sleep (i.e., the longest sleep bout) | Night | All | 1 | 0.03 |
| 117 | Sleep | Total time between midnight and the end time of the main sleep (i.e., the longest sleep bout) | Evening | Weekend | 1 | 0.03 |
| 118 | Sleep | Total time spent asleep | Evening | All | 1 | 0.01 |
| 119 | Sleep | Total sleep records/ bouts | Night | Weekend | 1 | 0.01 |
| 120 | Sleep | Total sleep records/ bouts | Evening | Weekend | 1 | 0 |
| 121 | Steps | Minutes labeled by Fitbit as 'sedentary' | Morning | All | 2 | 0.19 |
| 122 | Steps | Minutes labeled by Fitbit as 'lightly active' | Night | Weekday | 1 | 0.13 |
| 123 | Steps | Minutes labeled by Fitbit as 'fairly active' | Afternoon | Weekend | 3 | 0.13 |
| 124 | Steps | Minutes labeled by Fitbit as 'sedentary' | Evening | Weekday | 4 | 0.12 |
| 125 | Steps | Minutes labeled by Fitbit as 'sedentary' | Morning | Weekday | 1 | 0.12 |
| 126 | Steps | Minutes labeled by Fitbit as 'sedentary' | All | All | 1 | 0.12 |
| 127 | Steps | Minutes labeled by Fitbit as 'lightly active' | Morning | Weekend | 4 | 0.1 |
| 128 | Steps | Minutes labeled by Fitbit as 'lightly active' | Afternoon | Weekend | 2 | 0.1 |
| 129 | Steps | Total number of steps | Evening | Weekday | 1 | 0.1 |
| 130 | Steps | Minutes labeled by Fitbit as 'very active' | Morning | Weekday | 4 | 0.1 |
| 131 | Steps | Minutes labeled by Fitbit as 'lightly active' | Evening | Weekday | 3 | 0.08 |
| 132 | Steps | Minutes labeled by Fitbit as 'very active' | Afternoon | Weekend | 3 | 0.07 |
| 133 | Steps | Minutes labeled by Fitbit as 'lightly active' | Evening | Weekend | 2 | 0.07 |
| 134 | Steps | Minutes labeled by Fitbit as 'fairly active' | Evening | Weekend | 1 | 0.07 |
| 135 | Steps | Total number of steps | Afternoon | All | 2 | 0.07 |
| 136 | Steps | Total number of steps | All | Weekend | 3 | 0.07 |
| 137 | Steps | Minutes labeled by Fitbit as 'very active' | Evening | Weekend | 2 | 0.06 |
| 138 | Steps | Minutes labeled by Fitbit as 'very active' | Afternoon | Weekday | 1 | 0.05 |
| 139 | Steps | Minutes labeled by Fitbit as 'very active' | Night | All | 3 | 0.05 |
| 140 | Steps | Minutes labeled by Fitbit as 'very active' | Afternoon | All | 1 | 0.05 |
| 141 | Steps | Minutes labeled by Fitbit as 'fairly active' | Morning | Weekend | 3 | 0.05 |
| 142 | Steps | Total number of steps | Night | Weekend | 1 | 0.05 |
| 143 | Steps | Minutes labeled by Fitbit as 'lightly active' | Morning | All | 2 | 0.05 |
| 144 | Steps | Minutes labeled by Fitbit as 'very active' | Morning | Weekend | 5 | 0.04 |
| 145 | Steps | Total number of steps | Evening | Weekend | 4 | 0.03 |
| 146 | Steps | Minutes labeled by Fitbit as 'fairly active' | Evening | Weekday | 2 | 0.03 |
| 147 | Steps | Total number of steps | Night | All | 1 | 0.03 |
| 148 | Steps | Minutes labeled by Fitbit as 'fairly active' | Night | All | 3 | 0.03 |
| 149 | Steps | Minutes labeled by Fitbit as 'very active' | All | Weekday | 3 | 0.02 |
| 150 | Steps | Minutes labeled by Fitbit as 'very active' | All | Weekend | 1 | 0.02 |
| 151 | Steps | Minutes labeled by Fitbit as 'sedentary' | Night | Weekend | 3 | 0.01 |
| 152 | Steps | Minutes labeled by Fitbit as 'very active' | Night | Weekend | 2 | 0.01 |
| 153 | Steps | Minutes labeled by Fitbit as 'fairly active' | Night | Weekend | 1 | 0 |

Table S2 shows the features selected by the best 1-sensor models used to predict whether a person has high global MS symptom burden or low global MS symptom burden. The best model for global MS symptom burden contains calls, heart rate, location, and screen. Examples of the best features from these sensors (i.e., features with the highest absolute coefficients) include the change in duration of incoming calls during evenings on all days, change in time spent in the fat burn heart rate zone during afternoons on weekends, change in percentage of time spent at home during nights on weekends, and change in mean phone unlocks during afternoons on weekdays.

Table S2. Features selected by the best 1-sensor models when predicting high global MS symptom burden vs. low global MS symptom burden. SNO: Serial number.

| **SNO** | **Sensor** | **Feature** | **Time-of-the-Day** | **Days-of-the-Week** | **No. of Folds Selected** | **Mean Coefficient Across Folds** |
| --- | --- | --- | --- | --- | --- | --- |
| 1 | Calls | Duration of incoming calls | Evening | All | 5 | -0.79 |
| 2 | Calls | Number of outgoing calls | Morning | All | 3 | -0.5 |
| 3 | Calls | Duration of incoming calls | Evening | Weekday | 2 | -0.42 |
| 4 | Calls | Number of outgoing calls | Morning | Weekday | 1 | -0.4 |
| 5 | Calls | Number of incoming calls | Afternoon | All | 3 | -0.36 |
| 6 | Calls | Number of outgoing calls | Afternoon | Weekend | 5 | 0.36 |
| 7 | Calls | Number of incoming calls | Afternoon | Weekend | 1 | -0.29 |
| 8 | Calls | Number of missed calls | Evening | All | 1 | -0.28 |
| 9 | Calls | Duration of incoming calls | Afternoon | Weekday | 1 | 0.27 |
| 10 | Calls | Number of correspondents | Evening | All | 1 | 0.18 |
| 11 | Calls | Duration of incoming calls | Evening | Weekend | 1 | -0.16 |
| 12 | Calls | Number of correspondents | Morning | All | 1 | -0.1 |
| 13 | Calls | Duration of outgoing calls | Morning | All | 1 | 0 |
| 14 | Heart Rate | Time spent in the fat burn heart rate zone | Afternoon | Weekend | 3 | 0.13 |
| 15 | Heart Rate | Time spent in the fat burn heart rate zone | Evening | Weekday | 2 | 0.09 |
| 16 | Heart Rate | Average heart rate | Night | Weekend | 4 | 0.09 |
| 17 | Heart Rate | Average heart rate outside exercise heart rate zones (i.e., when person is at rest or has low activity) | All | Weekend | 4 | 0.08 |
| 18 | Heart Rate | Average heart rate | Afternoon | Weekend | 2 | 0.08 |
| 19 | Heart Rate | Time spent outside exercise heart rate zones (i.e., when person is at rest or has low activity) | Evening | Weekend | 3 | 0.07 |
| 20 | Heart Rate | Time spent outside exercise heart rate zones (i.e., when person is at rest or has low activity) | All | Weekend | 4 | 0.06 |
| 21 | Heart Rate | Time spent in the cardio heart rate zone | Afternoon | All | 1 | 0.06 |
| 22 | Heart Rate | Average heart rate outside exercise heart rate zones (i.e., when person is at rest or has low activity) | Evening | Weekend | 5 | 0.05 |
| 23 | Heart Rate | Average heart rate | Morning | Weekday | 1 | 0.05 |
| 24 | Heart Rate | Time spent outside exercise heart rate zones (i.e., when person is at rest or has low activity) | Night | Weekday | 2 | 0.05 |
| 25 | Heart Rate | Time spent in the cardio heart rate zone | All | Weekend | 4 | 0.05 |
| 26 | Heart Rate | Time spent in the cardio heart rate zone | Night | All | 4 | 0.05 |
| 27 | Heart Rate | Time spent in the cardio heart rate zone | Morning | Weekend | 4 | 0.03 |
| 28 | Heart Rate | Average heart rate outside exercise heart rate zones (i.e., when person is at rest or has low activity) | Afternoon | Weekend | 3 | 0.03 |
| 29 | Heart Rate | Average heart rate outside exercise heart rate zones (i.e., when person is at rest or has low activity) | Morning | Weekend | 3 | 0.03 |
| 30 | Heart Rate | Time spent in the fat burn heart rate zone | Night | Weekday | 2 | 0.03 |
| 31 | Heart Rate | Time spent in the cardio heart rate zone | Morning | Weekday | 4 | 0.03 |
| 32 | Heart Rate | Time spent outside exercise heart rate zones (i.e., when person is at rest or has low activity) | Evening | Weekday | 3 | 0.02 |
| 33 | Heart Rate | Average heart rate | All | Weekend | 3 | 0.02 |
| 34 | Heart Rate | Average heart rate outside exercise heart rate zones (i.e., when person is at rest or has low activity) | Morning | Weekday | 1 | 0.02 |
| 35 | Heart Rate | Time spent in the fat burn heart rate zone | Night | Weekend | 1 | 0.02 |
| 36 | Heart Rate | Time spent in the cardio heart rate zone | Night | Weekday | 3 | 0.02 |
| 37 | Heart Rate | Time spent in the cardio heart rate zone | Morning | All | 2 | 0.02 |
| 38 | Heart Rate | Time spent in the cardio heart rate zone | Afternoon | Weekday | 1 | 0.02 |
| 39 | Heart Rate | Average heart rate outside exercise heart rate zones (i.e., when person is at rest or has low activity) | Evening | All | 3 | 0.02 |
| 40 | Heart Rate | Time spent in the fat burn heart rate zone | Afternoon | Weekday | 1 | 0.02 |
| 41 | Heart Rate | Time spent in the fat burn heart rate zone | Evening | Weekend | 3 | 0.02 |
| 42 | Heart Rate | Average heart rate outside exercise heart rate zones (i.e., when person is at rest or has low activity) | All | Weekday | 1 | 0.02 |
| 43 | Heart Rate | Time spent in the cardio heart rate zone | Night | Weekend | 5 | 0.02 |
| 44 | Heart Rate | Average heart rate | Evening | Weekday | 1 | 0.02 |
| 45 | Heart Rate | Time spent in the cardio heart rate zone | All | Weekday | 4 | 0.02 |
| 46 | Heart Rate | Time spent outside exercise heart rate zones (i.e., when person is at rest or has low activity) | Night | All | 1 | 0.02 |
| 47 | Heart Rate | Time spent outside exercise heart rate zones (i.e., when person is at rest or has low activity) | Night | Weekend | 3 | 0.01 |
| 48 | Heart Rate | Time spent in the cardio heart rate zone | Afternoon | Weekend | 3 | 0.01 |
| 49 | Heart Rate | Time spent in the fat burn heart rate zone | Afternoon | All | 2 | 0.01 |
| 50 | Heart Rate | Time spent in the fat burn heart rate zone | Morning | Weekday | 2 | 0.01 |
| 51 | Heart Rate | Time spent in the cardio heart rate zone | Evening | Weekend | 3 | 0.01 |
| 52 | Heart Rate | Time spent in the fat burn heart rate zone | Evening | All | 2 | 0.01 |
| 53 | Heart Rate | Time spent in peak heart rate zone | Night | Weekend | 5 | 0.01 |
| 54 | Heart Rate | Time spent in the fat burn heart rate zone | All | All | 1 | 0.01 |
| 55 | Heart Rate | Average heart rate outside exercise heart rate zones (i.e., when person is at rest or has low activity) | Night | Weekend | 3 | 0.01 |
| 56 | Heart Rate | Average heart rate | Morning | All | 1 | 0.01 |
| 57 | Heart Rate | Time spent in peak heart rate zone | Afternoon | Weekend | 5 | 0.01 |
| 58 | Heart Rate | Time spent in peak heart rate zone | Morning | All | 2 | 0.01 |
| 59 | Heart Rate | Time spent in the cardio heart rate zone | Evening | Weekday | 3 | 0 |
| 60 | Heart Rate | Average heart rate | Afternoon | Weekday | 1 | 0 |
| 61 | Heart Rate | Average heart rate outside exercise heart rate zones (i.e., when person is at rest or has low activity) | Afternoon | Weekday | 1 | 0 |
| 62 | Heart Rate | Time spent in peak heart rate zone | Evening | Weekday | 4 | 0 |
| 63 | Heart Rate | Average heart rate outside exercise heart rate zones (i.e., when person is at rest or has low activity) | Evening | Weekday | 2 | 0 |
| 64 | Heart Rate | Average heart rate outside exercise heart rate zones (i.e., when person is at rest or has low activity) | Afternoon | All | 5 | 0 |
| 65 | Heart Rate | Time spent in the fat burn heart rate zone | Morning | Weekend | 2 | 0 |
| 66 | Heart Rate | Time spent in peak heart rate zone | Morning | Weekend | 3 | 0 |
| 67 | Heart Rate | Time spent in the fat burn heart rate zone | All | Weekend | 2 | 0 |
| 68 | Heart Rate | Time spent in the fat burn heart rate zone | All | Weekday | 1 | 0 |
| 69 | Heart Rate | Time spent in peak heart rate zone | All | Weekend | 2 | 0 |
| 70 | Heart Rate | Average heart rate | Morning | Weekend | 2 | 0 |
| 71 | Heart Rate | Time spent in peak heart rate zone | Morning | Weekday | 2 | 0 |
| 72 | Heart Rate | Time spent in the cardio heart rate zone | Evening | All | 1 | 0 |
| 73 | Heart Rate | Time spent outside exercise heart rate zones (i.e., when person is at rest or has low activity) | Morning | Weekend | 1 | 0 |
| 74 | Heart Rate | Time spent in peak heart rate zone | Night | Weekday | 4 | 0 |
| 75 | Heart Rate | Average heart rate outside exercise heart rate zones (i.e., when person is at rest or has low activity) | Morning | All | 1 | 0 |
| 76 | Heart Rate | Time spent in peak heart rate zone | Night | All | 4 | 0 |
| 77 | Heart Rate | Time spent in peak heart rate zone | Afternoon | Weekday | 1 | 0 |
| 78 | Heart Rate | Time spent in peak heart rate zone | Evening | Weekend | 5 | 0 |
| 79 | Heart Rate | Time spent outside exercise heart rate zones (i.e., when person is at rest or has low activity) | Afternoon | Weekend | 1 | 0 |
| 80 | Heart Rate | Time spent in the fat burn heart rate zone | Night | All | 1 | 0 |
| 81 | Heart Rate | Time spent in peak heart rate zone | Evening | All | 1 | 0 |
| 82 | Heart Rate | Average heart rate | Night | All | 1 | 0 |
| 83 | Heart Rate | Average heart rate | Night | Weekday | 1 | 0 |
| 84 | Location | Percentage of time spent at home | Night | Weekend | 1 | 1.06 |
| 85 | Location | Number of most frequented (significant) locations | All | Weekend | 5 | 0.96 |
| 86 | Location | Percentage of time spent at the second most frequented location | Afternoon | Weekday | 1 | -0.8 |
| 87 | Location | Percentage of time spent at rarely visited locations (e.g. new places) | Night | Weekday | 1 | -0.76 |
| 88 | Location | Percentage of time spent in motion (e.g. while driving, in public transit) | Night | Weekend | 3 | 0.75 |
| 89 | Location | Circadian movement i.e., regularity in movement patterns in 24 hr periods | Evening | Weekend | 1 | -0.74 |
| 90 | Location | Radius of the area covered during mobility | Evening | All | 3 | 0.65 |
| 91 | Location | Number of most frequented (significant) locations | Afternoon | Weekday | 2 | -0.63 |
| 92 | Location | Total distance traveled | All | Weekend | 4 | 0.59 |
| 93 | Location | Standard deviation of the duration of stay at most frequented (significant) locations | All | Weekend | 3 | -0.55 |
| 94 | Location | Average duration of stay at most frequented (significant) locations | Night | Weekend | 5 | -0.54 |
| 95 | Location | Average duration of stay at most frequented (significant) locations | Evening | All | 2 | 0.53 |
| 96 | Location | Standard deviation of the duration of stay at most frequented (significant) locations | Afternoon | Weekday | 1 | -0.49 |
| 97 | Location | Percentage of time spent at rarely visited locations (e.g. new places) | Evening | Weekend | 1 | -0.47 |
| 98 | Location | Percentage of time spent at rarely visited locations (e.g. new places) | Morning | Weekday | 3 | -0.45 |
| 99 | Location | Percentage of time spent at the third most frequented location | Evening | Weekend | 1 | -0.42 |
| 100 | Location | Average duration of stay at most frequented (significant) locations | Evening | Weekday | 1 | 0.37 |
| 101 | Location | Number of most frequented (significant) locations | Night | Weekend | 3 | 0.36 |
| 102 | Location | Total distance traveled | Evening | All | 1 | 0.34 |
| 103 | Location | Location entropy i.e., evenness in time spent across most frequented (significant) locations | Morning | Weekend | 3 | 0.32 |
| 104 | Location | Percentage of time spent at home | Morning | Weekday | 1 | -0.2 |
| 105 | Location | Number of most frequented (significant) locations | Morning | Weekend | 1 | 0.06 |
| 106 | Location | Percentage of time spent in motion (e.g. while driving, in public transit) | Evening | Weekday | 1 | 0.04 |
| 107 | Location | Standard deviation of the duration of stay at most frequented (significant) locations | Evening | Weekend | 2 | 0 |
| 108 | Location | Circadian movement i.e., regularity in movement patterns in 24 hr periods | Night | Weekday | 1 | 0 |
| 109 | Location | Percentage of time spent in motion (e.g. while driving, in public transit) | Morning | Weekday | 1 | 0 |
| 110 | Screen | Mean unlocks per minute | Afternoon | Weekday | 5 | 0.46 |
| 111 | Screen | Total time spent interacting with the phone | Evening | Weekday | 1 | 0 |
| 112 | Sleep | Total times restless during the main sleep (i.e., the longest sleep bout) | Night | Weekday | 1 | 0.25 |
| 113 | Sleep | Total time spent in bed during the main sleep (i.e., the longest sleep bout) | Morning | Weekday | 1 | 0.15 |
| 114 | Sleep | Total times restless during the main sleep (i.e., the longest sleep bout) | Morning | Weekday | 5 | 0.14 |
| 115 | Sleep | Total time between midnight and the end time of the main sleep (i.e., the longest sleep bout) | Evening | Weekday | 2 | 0.14 |
| 116 | Sleep | Total times restless during the main sleep (i.e., the longest sleep bout) | Evening | Weekend | 2 | 0.14 |
| 117 | Sleep | Total sleep records/ bouts | Evening | All | 2 | 0.13 |
| 118 | Sleep | Total sleep records/ bouts | Morning | All | 1 | 0.12 |
| 119 | Sleep | Total sleep records/ bouts | All | Weekend | 1 | 0.12 |
| 120 | Sleep | Total sleep records/ bouts | Night | All | 1 | 0.1 |
| 121 | Sleep | Sleep efficiency (time asleep / (total time in bed - time to fall asleep)) during the main sleep (i.e., the longest sleep bout) | All | Weekday | 3 | 0.1 |
| 122 | Sleep | Total duration restless during the main sleep (i.e., the longest sleep bout) | Morning | Weekday | 2 | 0.1 |
| 123 | Sleep | Total sleep records/ bouts | All | Weekday | 1 | 0.1 |
| 124 | Sleep | Sleep efficiency (time asleep / (total time in bed - time to fall asleep)) during the main sleep (i.e., the longest sleep bout) | Morning | Weekend | 4 | 0.08 |
| 125 | Sleep | Total time spent in bed | All | Weekday | 4 | 0.08 |
| 126 | Sleep | Total duration restless during the main sleep (i.e., the longest sleep bout) | Morning | All | 1 | 0.07 |
| 127 | Sleep | Total time between midnight and the end time of the main sleep (i.e., the longest sleep bout) | Night | Weekend | 1 | 0.07 |
| 128 | Sleep | Total time spent in bed during the main sleep (i.e., the longest sleep bout) | Morning | Weekend | 1 | 0.07 |
| 129 | Sleep | Total sleep records/ bouts | Evening | Weekday | 1 | 0.07 |
| 130 | Sleep | Total time between midnight and the end time of the main sleep (i.e., the longest sleep bout) | Evening | Weekend | 1 | 0.06 |
| 131 | Sleep | Total time between midnight and the start time of the main sleep (i.e., the longest sleep bout) | Night | Weekday | 1 | 0.06 |
| 132 | Sleep | Total duration restless during the main sleep (i.e., the longest sleep bout) | Evening | Weekday | 2 | 0.05 |
| 133 | Sleep | Total duration restless during the main sleep (i.e., the longest sleep bout) | Night | Weekend | 1 | 0.05 |
| 134 | Sleep | Sleep efficiency (time asleep / (total time in bed - time to fall asleep)) during the main sleep (i.e., the longest sleep bout) | All | All | 1 | 0.05 |
| 135 | Sleep | Sleep efficiency (time asleep / (total time in bed - time to fall asleep)) during the main sleep (i.e., the longest sleep bout) | Evening | Weekend | 3 | 0.04 |
| 136 | Sleep | Total sleep records/ bouts | Night | Weekday | 3 | 0.04 |
| 137 | Sleep | Total sleep records/ bouts | Night | Weekend | 5 | 0.04 |
| 138 | Sleep | Total time between midnight and the start time of the main sleep (i.e., the longest sleep bout) | Morning | All | 1 | 0.04 |
| 139 | Sleep | Total time between midnight and the start time of the main sleep (i.e., the longest sleep bout) | Morning | Weekend | 1 | 0.04 |
| 140 | Sleep | Total time spent asleep | All | Weekend | 4 | 0.03 |
| 141 | Sleep | Total time spent asleep | Night | Weekend | 1 | 0.03 |
| 142 | Sleep | Total sleep records/ bouts | Morning | Weekend | 1 | 0.02 |
| 143 | Sleep | Time spent asleep during the main sleep (i.e., the longest sleep bout) | Evening | Weekday | 1 | 0.01 |
| 144 | Sleep | Total sleep records/ bouts | Evening | Weekend | 1 | 0.01 |
| 145 | Steps | Minutes labeled by Fitbit as 'lightly active' | All | Weekday | 1 | 0.43 |
| 146 | Steps | Minutes labeled by Fitbit as 'sedentary' | Night | Weekday | 1 | 0.41 |
| 147 | Steps | Minutes labeled by Fitbit as 'very active' | All | Weekend | 1 | 0.24 |
| 148 | Steps | Minutes labeled by Fitbit as 'very active' | All | Weekday | 2 | 0.23 |
| 149 | Steps | Minutes labeled by Fitbit as 'very active' | Morning | Weekend | 1 | 0.21 |
| 150 | Steps | Minutes labeled by Fitbit as 'fairly active' | All | Weekday | 1 | 0.21 |
| 151 | Steps | Total number of steps | Afternoon | Weekend | 3 | 0.17 |
| 152 | Steps | Minutes labeled by Fitbit as 'fairly active' | Night | All | 5 | 0.17 |
| 153 | Steps | Minutes labeled by Fitbit as 'fairly active' | Afternoon | Weekend | 1 | 0.15 |
| 154 | Steps | Minutes labeled by Fitbit as 'lightly active' | Morning | Weekend | 1 | 0.13 |
| 155 | Steps | Minutes labeled by Fitbit as 'very active' | Night | All | 2 | 0.12 |
| 156 | Steps | Minutes labeled by Fitbit as 'very active' | All | All | 2 | 0.12 |
| 157 | Steps | Minutes labeled by Fitbit as 'very active' | Evening | All | 3 | 0.11 |
| 158 | Steps | Minutes labeled by Fitbit as 'very active' | Evening | Weekend | 4 | 0.1 |
| 159 | Steps | Total number of steps | All | Weekend | 1 | 0.08 |
| 160 | Steps | Minutes labeled by Fitbit as 'fairly active' | Afternoon | All | 1 | 0.07 |
| 161 | Steps | Minutes labeled by Fitbit as 'fairly active' | All | All | 1 | 0.02 |
| 162 | Steps | Minutes labeled by Fitbit as 'fairly active' | Night | Weekend | 1 | 0.01 |
| 163 | Steps | Minutes labeled by Fitbit as 'very active' | Night | Weekend | 1 | 0 |

Table S3 shows the features selected by the best 1-sensor models used to predict whether a person has severe fatigue or low fatigue. The best model for fatigue contains calls, heart rate, and location. Examples of the best features from these sensors (*i.e.*, features with the highest absolute coefficients) include the change in number of outgoing calls during mornings on all days, change in time spent in the cardio heart rate zone during mornings on weekends, and change in Location entropy, *i.e.*, evenness in time spent across most frequented (significant) locations during mornings on all days.

Table S3. Features selected by the best 1-sensor models when predicting severe fatigue vs. low fatigue. SNO: Serial number.

| **SNO** | **Sensor** | **Feature** | **Time-of-the-Day** | **Days-of-the-Week** | **No. of Folds Selected** | **Mean Coefficient Across Folds** |
| --- | --- | --- | --- | --- | --- | --- |
| 1 | Calls | Number of outgoing calls | Morning | All | 1 | 0.27 |
| 2 | Calls | Duration of incoming calls | Evening | Weekday | 5 | 0.24 |
| 3 | Calls | Number of outgoing calls | Evening | Weekday | 1 | 0.17 |
| 4 | Calls | Number of incoming calls | Afternoon | All | 5 | 0.16 |
| 5 | Calls | Number of incoming calls | Evening | All | 5 | 0.12 |
| 6 | Calls | Duration of incoming calls | Morning | Weekday | 1 | 0.11 |
| 7 | Calls | Number of missed calls | Evening | All | 1 | 0.09 |
| 8 | Calls | Duration of outgoing calls | Morning | All | 5 | 0.06 |
| 9 | Calls | Number of incoming calls | Afternoon | Weekday | 4 | 0.05 |
| 10 | Calls | Number of incoming calls | Evening | Weekday | 1 | 0.05 |
| 11 | Calls | Number of outgoing calls | Afternoon | Weekend | 5 | 0.03 |
| 12 | Calls | Number of correspondents | Morning | Weekend | 4 | 0.03 |
| 13 | Calls | Duration of incoming calls | Afternoon | Weekday | 5 | 0.03 |
| 14 | Calls | Number of incoming calls | Afternoon | Weekend | 1 | 0.02 |
| 15 | Calls | Duration of incoming calls | Afternoon | All | 2 | 0.02 |
| 16 | Calls | Number of incoming calls | Morning | Weekend | 1 | 0.01 |
| 17 | Calls | Duration of outgoing calls | Morning | Weekend | 1 | 0 |
| 18 | Calls | Number of outgoing calls | Evening | All | 1 | 0 |
| 19 | Calls | Duration of incoming calls | Evening | Weekend | 1 | 0 |
| 20 | Calls | Number of outgoing calls | All | Weekend | 1 | 0 |
| 21 | Calls | Number of outgoing calls | Afternoon | Weekday | 1 | 0 |
| 22 | Heart Rate | Time spent in the cardio heart rate zone | Morning | Weekend | 5 | -0.88 |
| 23 | Heart Rate | Time spent in the cardio heart rate zone | Morning | Weekday | 5 | 0.75 |
| 24 | Heart Rate | Average heart rate outside exercise heart rate zones (i.e., when person is at rest or has low activity) | All | Weekend | 5 | 0.59 |
| 25 | Heart Rate | Average heart rate outside exercise heart rate zones (i.e., when person is at rest or has low activity) | Evening | Weekend | 5 | -0.53 |
| 26 | Heart Rate | Time spent outside exercise heart rate zones (i.e., when person is at rest or has low activity) | Morning | Weekend | 5 | -0.52 |
| 27 | Heart Rate | Time spent in peak heart rate zone | Afternoon | Weekend | 5 | 0.44 |
| 28 | Heart Rate | Time spent in peak heart rate zone | Evening | Weekend | 5 | -0.44 |
| 29 | Heart Rate | Time spent in the fat burn heart rate zone | Night | Weekday | 4 | 0.28 |
| 30 | Heart Rate | Time spent in peak heart rate zone | Morning | Weekend | 4 | 0.25 |
| 31 | Heart Rate | Time spent in the fat burn heart rate zone | Night | All | 5 | 0.23 |
| 32 | Heart Rate | Average heart rate | Evening | Weekday | 2 | -0.22 |
| 33 | Heart Rate | Time spent in the fat burn heart rate zone | Night | Weekend | 2 | 0.22 |
| 34 | Heart Rate | Time spent outside exercise heart rate zones (i.e., when person is at rest or has low activity) | All | Weekend | 5 | -0.22 |
| 35 | Heart Rate | Average heart rate outside exercise heart rate zones (i.e., when person is at rest or has low activity) | Morning | Weekend | 4 | 0.21 |
| 36 | Heart Rate | Time spent outside exercise heart rate zones (i.e., when person is at rest or has low activity) | Night | All | 2 | -0.18 |
| 37 | Heart Rate | Average heart rate | Evening | All | 2 | -0.18 |
| 38 | Heart Rate | Time spent in peak heart rate zone | Morning | All | 1 | 0.14 |
| 39 | Heart Rate | Time spent in peak heart rate zone | Night | Weekend | 5 | 0.12 |
| 40 | Heart Rate | Time spent outside exercise heart rate zones (i.e., when person is at rest or has low activity) | Evening | Weekend | 3 | 0.09 |
| 41 | Heart Rate | Time spent in the fat burn heart rate zone | All | Weekend | 5 | -0.09 |
| 42 | Heart Rate | Average heart rate | Afternoon | Weekend | 3 | -0.09 |
| 43 | Heart Rate | Average heart rate outside exercise heart rate zones (i.e., when person is at rest or has low activity) | Evening | All | 4 | -0.09 |
| 44 | Heart Rate | Time spent outside exercise heart rate zones (i.e., when person is at rest or has low activity) | Night | Weekend | 3 | 0.09 |
| 45 | Heart Rate | Time spent outside exercise heart rate zones (i.e., when person is at rest or has low activity) | All | All | 3 | -0.08 |
| 46 | Heart Rate | Time spent outside exercise heart rate zones (i.e., when person is at rest or has low activity) | Night | Weekday | 5 | -0.07 |
| 47 | Heart Rate | Time spent in the cardio heart rate zone | Night | Weekend | 5 | 0.07 |
| 48 | Heart Rate | Time spent in the cardio heart rate zone | Evening | Weekend | 3 | -0.05 |
| 49 | Heart Rate | Time spent in the fat burn heart rate zone | Evening | All | 1 | -0.05 |
| 50 | Heart Rate | Time spent in the fat burn heart rate zone | Evening | Weekend | 4 | -0.05 |
| 51 | Heart Rate | Time spent outside exercise heart rate zones (i.e., when person is at rest or has low activity) | Morning | All | 3 | -0.05 |
| 52 | Heart Rate | Average heart rate outside exercise heart rate zones (i.e., when person is at rest or has low activity) | Afternoon | Weekend | 3 | -0.03 |
| 53 | Heart Rate | Time spent in peak heart rate zone | Afternoon | Weekday | 3 | -0.02 |
| 54 | Heart Rate | Average heart rate | Night | Weekend | 4 | 0.02 |
| 55 | Heart Rate | Time spent in peak heart rate zone | All | Weekend | 5 | 0 |
| 56 | Heart Rate | Time spent in the cardio heart rate zone | All | Weekend | 4 | 0 |
| 57 | Heart Rate | Time spent in peak heart rate zone | Evening | Weekday | 3 | 0 |
| 58 | Heart Rate | Time spent in the cardio heart rate zone | Morning | All | 3 | 0 |
| 59 | Heart Rate | Time spent outside exercise heart rate zones (i.e., when person is at rest or has low activity) | Afternoon | Weekend | 3 | 0 |
| 60 | Heart Rate | Time spent in peak heart rate zone | Night | All | 2 | 0 |
| 61 | Heart Rate | Time spent in peak heart rate zone | Night | Weekday | 2 | 0 |
| 62 | Heart Rate | Average heart rate | Morning | All | 2 | 0 |
| 63 | Heart Rate | Average heart rate | Morning | Weekend | 2 | 0 |
| 64 | Heart Rate | Time spent in the fat burn heart rate zone | Afternoon | Weekday | 2 | 0 |
| 65 | Heart Rate | Time spent in the fat burn heart rate zone | Afternoon | Weekend | 2 | 0 |
| 66 | Heart Rate | Time spent in the fat burn heart rate zone | Evening | Weekday | 1 | 0 |
| 67 | Heart Rate | Time spent outside exercise heart rate zones (i.e., when person is at rest or has low activity) | Evening | Weekday | 1 | 0 |
| 68 | Heart Rate | Average heart rate outside exercise heart rate zones (i.e., when person is at rest or has low activity) | Evening | Weekday | 1 | 0 |
| 69 | Heart Rate | Time spent in the cardio heart rate zone | Night | All | 1 | 0 |
| 70 | Heart Rate | Average heart rate outside exercise heart rate zones (i.e., when person is at rest or has low activity) | Night | All | 1 | 0 |
| 71 | Heart Rate | Average heart rate | Night | Weekday | 1 | 0 |
| 72 | Heart Rate | Average heart rate outside exercise heart rate zones (i.e., when person is at rest or has low activity) | Night | Weekday | 1 | 0 |
| 73 | Heart Rate | Average heart rate outside exercise heart rate zones (i.e., when person is at rest or has low activity) | Night | Weekend | 1 | 0 |
| 74 | Heart Rate | Average heart rate outside exercise heart rate zones (i.e., when person is at rest or has low activity) | All | All | 1 | 0 |
| 75 | Heart Rate | Time spent in the cardio heart rate zone | All | Weekday | 1 | 0 |
| 76 | Heart Rate | Time spent outside exercise heart rate zones (i.e., when person is at rest or has low activity) | All | Weekday | 1 | 0 |
| 77 | Heart Rate | Average heart rate outside exercise heart rate zones (i.e., when person is at rest or has low activity) | All | Weekday | 1 | 0 |
| 78 | Heart Rate | Time spent in peak heart rate zone | Morning | Weekday | 1 | 0 |
| 79 | Heart Rate | Average heart rate | Morning | Weekday | 1 | 0 |
| 80 | Heart Rate | Time spent in the fat burn heart rate zone | Morning | Weekend | 1 | 0 |
| 81 | Heart Rate | Average heart rate outside exercise heart rate zones (i.e., when person is at rest or has low activity) | Afternoon | Weekday | 1 | 0 |
| 82 | Heart Rate | Time spent in the cardio heart rate zone | Afternoon | Weekend | 1 | 0 |
| 83 | Location | Location entropy i.e., evenness in time spent across most frequented (significant) locations | Morning | All | 4 | 0.23 |
| 84 | Location | Standard deviation of the duration of stay at most frequented (significant) locations | All | Weekday | 4 | 0.19 |
| 85 | Location | Percentage of time spent at the most frequented (primary) location | All | Weekend | 1 | 0.15 |
| 86 | Location | Location entropy i.e., evenness in time spent across most frequented (significant) locations | Morning | Weekend | 3 | 0.15 |
| 87 | Location | Radius of the area covered during mobility | Afternoon | Weekend | 4 | 0.14 |
| 88 | Location | Standard deviation of the duration of stay at most frequented (significant) locations | Evening | Weekend | 1 | 0.1 |
| 89 | Location | Standard deviation of the duration of stay at most frequented (significant) locations | Afternoon | Weekend | 2 | 0.1 |
| 90 | Location | Standard deviation of the duration of stay at most frequented (significant) locations | All | All | 4 | 0.09 |
| 91 | Location | Percentage of time spent at the most frequented (primary) location | Afternoon | Weekend | 1 | 0.09 |
| 92 | Location | Standard deviation of the duration of stay at most frequented (significant) locations | Evening | Weekday | 1 | 0.06 |
| 93 | Location | Percentage of time spent at the third most frequented location | Evening | Weekend | 1 | 0.05 |
| 94 | Location | Percentage of time spent at the second most frequented location | Night | Weekend | 1 | 0.04 |
| 95 | Location | Number of most frequented (significant) locations | Morning | All | 2 | 0.04 |
| 96 | Location | Normalized location entropy i.e., evenness in time spent across most frequented (significant) locations | Morning | Weekend | 2 | 0.03 |
| 97 | Location | Percentage of time spent at the second most frequented location | Morning | Weekend | 1 | 0.03 |
| 98 | Location | Number of most frequented (significant) locations | Morning | Weekday | 2 | 0.03 |
| 99 | Location | Radius of the area covered during mobility | Evening | All | 3 | 0.03 |
| 100 | Location | Percentage of time spent at home | Night | All | 4 | 0.03 |
| 101 | Location | Normalized location entropy i.e., evenness in time spent across most frequented (significant) locations | Evening | All | 1 | 0.02 |
| 102 | Location | Number of most frequented (significant) locations | All | Weekend | 2 | 0.02 |
| 103 | Location | Percentage of time spent in motion (e.g. while driving, in public transit) | Evening | All | 1 | 0.02 |
| 104 | Location | Normalized location entropy i.e., evenness in time spent across most frequented (significant) locations | All | Weekday | 1 | 0.02 |
| 105 | Location | Percentage of time spent at home | Night | Weekday | 1 | 0.01 |
| 106 | Location | Number of most frequented (significant) locations | Morning | Weekend | 1 | 0.01 |
| 107 | Location | Average duration of stay at most frequented (significant) locations | Evening | All | 1 | 0.01 |
| 108 | Location | Normalized location entropy i.e., evenness in time spent across most frequented (significant) locations | All | All | 1 | 0.01 |
| 109 | Location | Average duration of stay at most frequented (significant) locations | Afternoon | All | 1 | 0 |
| 110 | Location | Circadian movement i.e., regularity in movement patterns in 24 hr periods | Afternoon | Weekday | 1 | 0 |
| 111 | Location | Average duration of stay at most frequented (significant) locations | Morning | Weekday | 1 | 0 |
| 112 | Location | Location variance | Evening | All | 1 | 0 |
| 113 | Location | Location entropy i.e., evenness in time spent across most frequented (significant) locations | All | Weekend | 1 | 0 |
| 114 | Location | Percentage of time spent at home | Morning | Weekday | 1 | 0 |
| 115 | Location | Location entropy i.e., evenness in time spent across most frequented (significant) locations | Morning | Weekday | 1 | 0 |
| 116 | Screen | Number of unlocks | Afternoon | Weekday | 1 | 0.57 |
| 117 | Screen | Mean unlocks per minute | All | Weekday | 1 | 0.51 |
| 118 | Screen | Total time spent interacting with the phone | Night | All | 1 | 0.48 |
| 119 | Screen | Number of unlocks | Night | Weekend | 1 | 0.41 |
| 120 | Screen | Mean unlocks per minute | Afternoon | All | 1 | 0.38 |
| 121 | Screen | Mean unlocks per minute | Afternoon | Weekday | 2 | 0.33 |
| 122 | Screen | Total time spent interacting with the phone | All | Weekday | 2 | 0.28 |
| 123 | Screen | Total time spent interacting with the phone | Evening | Weekday | 3 | 0.23 |
| 124 | Screen | Total time spent interacting with the phone | Night | Weekend | 1 | 0.21 |
| 125 | Screen | Median time spent interacting with the phone per interaction or use | Afternoon | Weekday | 1 | 0.2 |
| 126 | Screen | Total time spent interacting with the phone | Morning | Weekday | 1 | 0.18 |
| 127 | Screen | Mean unlocks per minute | All | All | 1 | 0.15 |
| 128 | Sleep | Total times restless during the main sleep (i.e., the longest sleep bout) | Night | All | 1 | 1 |
| 129 | Sleep | Total time between midnight and the end time of the main sleep (i.e., the longest sleep bout) | Night | Weekday | 1 | 1 |
| 130 | Sleep | Total times restless during the main sleep (i.e., the longest sleep bout) | All | Weekend | 1 | 0.67 |
| 131 | Sleep | Sleep efficiency (time asleep / (total time in bed - time to fall asleep)) during the main sleep (i.e., the longest sleep bout) | Evening | Weekday | 1 | 0.51 |
| 132 | Sleep | Total time between midnight and the end time of the main sleep (i.e., the longest sleep bout) | Evening | All | 1 | 0.46 |
| 133 | Sleep | Total times restless during the main sleep (i.e., the longest sleep bout) | Morning | All | 1 | 0.31 |
| 134 | Sleep | Total sleep records/ bouts | Evening | All | 1 | 0.27 |
| 135 | Sleep | Total sleep records/ bouts | Night | All | 3 | 0.26 |
| 136 | Steps | Total number of steps | All | Weekday | 1 | 0.33 |
| 137 | Steps | Total number of steps | Night | Weekday | 3 | 0.23 |
| 138 | Steps | Minutes labeled by Fitbit as 'fairly active' | Evening | Weekend | 1 | 0.17 |
| 139 | Steps | Minutes labeled by Fitbit as 'fairly active' | Afternoon | Weekday | 1 | 0.17 |
| 140 | Steps | Total number of steps | All | All | 1 | 0.13 |
| 141 | Steps | Minutes labeled by Fitbit as 'lightly active' | Morning | All | 1 | 0.13 |
| 142 | Steps | Total number of steps | All | Weekend | 1 | 0.12 |
| 143 | Steps | Minutes labeled by Fitbit as 'sedentary' | Night | Weekend | 1 | 0.1 |
| 144 | Steps | Minutes labeled by Fitbit as 'very active' | All | Weekday | 4 | 0.09 |
| 145 | Steps | Minutes labeled by Fitbit as 'lightly active' | Night | Weekend | 2 | 0.09 |
| 146 | Steps | Minutes labeled by Fitbit as 'very active' | All | Weekend | 3 | 0.09 |
| 147 | Steps | Minutes labeled by Fitbit as 'fairly active' | Afternoon | All | 1 | 0.08 |
| 148 | Steps | Minutes labeled by Fitbit as 'fairly active' | Night | Weekday | 5 | 0.08 |
| 149 | Steps | Minutes labeled by Fitbit as 'sedentary' | Morning | All | 1 | 0.08 |
| 150 | Steps | Minutes labeled by Fitbit as 'fairly active' | Morning | Weekend | 3 | 0.08 |
| 151 | Steps | Minutes labeled by Fitbit as 'fairly active' | Night | All | 5 | 0.08 |
| 152 | Steps | Minutes labeled by Fitbit as 'sedentary' | Afternoon | Weekend | 1 | 0.07 |
| 153 | Steps | Minutes labeled by Fitbit as 'lightly active' | Morning | Weekend | 1 | 0.06 |
| 154 | Steps | Minutes labeled by Fitbit as 'sedentary' | Afternoon | All | 1 | 0.05 |
| 155 | Steps | Minutes labeled by Fitbit as 'very active' | Morning | Weekday | 4 | 0.04 |
| 156 | Steps | Minutes labeled by Fitbit as 'lightly active' | Afternoon | Weekend | 2 | 0.04 |
| 157 | Steps | Minutes labeled by Fitbit as 'lightly active' | Afternoon | All | 1 | 0.03 |
| 158 | Steps | Minutes labeled by Fitbit as 'lightly active' | Afternoon | Weekday | 1 | 0.03 |
| 159 | Steps | Minutes labeled by Fitbit as 'very active' | All | All | 5 | 0.02 |
| 160 | Steps | Minutes labeled by Fitbit as 'very active' | Night | All | 1 | 0.02 |
| 161 | Steps | Minutes labeled by Fitbit as 'fairly active' | Morning | Weekday | 1 | 0.02 |
| 162 | Steps | Minutes labeled by Fitbit as 'sedentary' | Evening | Weekday | 1 | 0.01 |
| 163 | Steps | Minutes labeled by Fitbit as 'sedentary' | All | Weekend | 1 | 0.01 |
| 164 | Steps | Minutes labeled by Fitbit as 'sedentary' | Morning | Weekday | 1 | 0.01 |
| 165 | Steps | Minutes labeled by Fitbit as 'very active' | Afternoon | Weekend | 3 | 0.01 |
| 166 | Steps | Minutes labeled by Fitbit as 'very active' | Morning | All | 1 | 0.01 |
| 167 | Steps | Minutes labeled by Fitbit as 'sedentary' | Evening | All | 1 | 0.01 |
| 168 | Steps | Minutes labeled by Fitbit as 'fairly active' | All | Weekday | 1 | 0 |
| 169 | Steps | Minutes labeled by Fitbit as 'very active' | Night | Weekend | 1 | 0 |
| 170 | Steps | Minutes labeled by Fitbit as 'lightly active' | All | Weekday | 1 | 0 |
| 171 | Steps | Total number of steps | Evening | Weekend | 1 | 0 |

Table S4 shows the features selected by the best 1-sensor models used to predict whether a person has poor sleep quality or better sleep quality. The best model for sleep quality contains location and screen. Examples of the best features from these sensors (*i.e.*, features with the highest absolute coefficients) include the change in circadian movement, *i.e.*, regularity in movement patterns in 24-hour periods during nights on all days, and change in mean unlocks per minute during nights on weekdays.

*Table S4. Features selected by the best 1-sensor models when predicting poor sleep quality vs. better sleep quality*. *SNO: Serial number.*

| **SNO** | **Sensor** | **Feature** | **Time-of-the-Day** | **Days-of-the-Week** | **No. of Folds Selected In** | **Mean Coefficient Across Folds** |
| --- | --- | --- | --- | --- | --- | --- |
| 1 | Calls | Number of outgoing calls | Morning | All | 1 | -0.55 |
| 2 | Calls | Duration of incoming calls | All | Weekend | 5 | -0.54 |
| 3 | Calls | Number of incoming calls | Afternoon | All | 1 | -0.5 |
| 4 | Calls | Number of missed calls | Evening | All | 1 | 0.45 |
| 5 | Calls | Number of correspondents | Afternoon | Weekend | 4 | -0.44 |
| 6 | Calls | Duration of outgoing calls | Afternoon | Weekday | 2 | 0.4 |
| 7 | Calls | Number of correspondents | Afternoon | All | 1 | -0.32 |
| 8 | Calls | Number of missed calls | All | Weekend | 1 | -0.3 |
| 9 | Calls | Duration of outgoing calls | All | Weekend | 2 | -0.28 |
| 10 | Calls | Number of correspondents | All | Weekend | 2 | -0.27 |
| 11 | Calls | Number of outgoing calls | Afternoon | Weekend | 3 | 0.26 |
| 12 | Calls | Number of incoming calls | Afternoon | Weekend | 1 | -0.26 |
| 13 | Calls | Number of missed calls | Evening | Weekend | 2 | 0.22 |
| 14 | Calls | Number of outgoing calls | Evening | Weekday | 1 | -0.22 |
| 15 | Calls | Number of correspondents | Morning | Weekend | 2 | -0.21 |
| 16 | Calls | Duration of incoming calls | Evening | All | 2 | -0.19 |
| 17 | Calls | Duration of incoming calls | Afternoon | Weekday | 1 | 0.17 |
| 18 | Calls | Number of incoming calls | Morning | Weekend | 2 | 0.15 |
| 19 | Calls | Number of outgoing calls | Morning | Weekday | 1 | -0.12 |
| 20 | Calls | Number of missed calls | Afternoon | Weekend | 1 | -0.06 |
| 21 | Calls | Duration of incoming calls | Morning | All | 2 | 0 |
| 22 | Calls | Duration of outgoing calls | Evening | All | 1 | 0 |
| 23 | Heart Rate | Time spent in the cardio heart rate zone | Afternoon | Weekend | 4 | 0.5 |
| 24 | Heart Rate | Time spent outside exercise heart rate zones (i.e., when person is at rest or has low activity) | Night | All | 3 | 0.49 |
| 25 | Heart Rate | Time spent outside exercise heart rate zones (i.e., when person is at rest or has low activity) | Night | Weekday | 1 | 0.36 |
| 26 | Heart Rate | Average heart rate outside exercise heart rate zones (i.e., when person is at rest or has low activity) | All | Weekday | 1 | 0.36 |
| 27 | Heart Rate | Time spent in peak heart rate zone | Afternoon | All | 1 | 0.18 |
| 28 | Heart Rate | Time spent in peak heart rate zone | Evening | All | 1 | 0.16 |
| 29 | Heart Rate | Time spent in peak heart rate zone | All | Weekend | 3 | 0.14 |
| 30 | Heart Rate | Time spent in peak heart rate zone | Evening | Weekend | 1 | 0.06 |
| 31 | Location | Circadian movement i.e., regularity in movement patterns in 24 hr periods | Night | All | 1 | 0.32 |
| 32 | Location | Percentage of time spent in motion (e.g. while driving, in public transit) | Morning | Weekend | 1 | 0.23 |
| 33 | Location | Percentage of time spent at rarely visited locations (e.g. new places) | Night | Weekday | 4 | 0.21 |
| 34 | Location | Percentage of time spent at rarely visited locations (e.g. new places) | Morning | Weekend | 4 | 0.16 |
| 35 | Location | Average duration of stay at most frequented (significant) locations | All | Weekday | 1 | 0.14 |
| 36 | Location | Total distance traveled | All | Weekend | 5 | 0.13 |
| 37 | Location | Location entropy i.e., evenness in time spent across most frequented (significant) locations | All | Weekend | 3 | 0.13 |
| 38 | Location | Circadian movement i.e., regularity in movement patterns in 24 hr periods | Evening | Weekend | 3 | 0.1 |
| 39 | Location | Standard deviation of the duration of stay at most frequented (significant) locations | Night | All | 1 | 0.1 |
| 40 | Location | Percentage of time spent at the second most frequented location | Evening | Weekend | 2 | 0.08 |
| 41 | Location | Percentage of time spent at home | Morning | Weekend | 1 | 0.08 |
| 42 | Location | Circadian movement i.e., regularity in movement patterns in 24 hr periods | Afternoon | Weekday | 1 | 0.06 |
| 43 | Location | Location variance | All | Weekend | 2 | 0.04 |
| 44 | Location | Location entropy i.e., evenness in time spent across most frequented (significant) locations | Morning | Weekend | 3 | 0.04 |
| 45 | Location | Number of most frequented (significant) locations | Evening | All | 3 | 0.02 |
| 46 | Location | Location variance | Afternoon | Weekday | 1 | 0.02 |
| 47 | Location | Standard deviation of the duration of stay at most frequented (significant) locations | Evening | Weekend | 2 | 0.01 |
| 48 | Location | Circadian movement i.e., regularity in movement patterns in 24 hr periods | Night | Weekday | 1 | 0 |
| 49 | Location | Circadian movement i.e., regularity in movement patterns in 24 hr periods | Morning | Weekday | 1 | 0 |
| 50 | Location | Number of most frequented (significant) locations | Morning | All | 1 | 0 |
| 51 | Screen | Mean unlocks per minute | Night | Weekday | 5 | 0.63 |
| 52 | Screen | Total time spent interacting with the phone | Afternoon | Weekday | 1 | 0.56 |
| 53 | Screen | Number of unlocks | Morning | Weekday | 1 | 0.51 |
| 54 | Screen | Median time spent interacting with the phone per interaction or use | All | Weekend | 1 | 0.44 |
| 55 | Screen | Number of unlocks | Night | Weekend | 1 | 0.32 |
| 56 | Sleep | Total duration restless during the main sleep (i.e., the longest sleep bout) | All | Weekday | 1 | 0.68 |
| 57 | Sleep | Total times restless during the main sleep (i.e., the longest sleep bout) | All | Weekday | 4 | 0.63 |
| 58 | Sleep | Total times restless during the main sleep (i.e., the longest sleep bout) | Evening | All | 1 | 0.58 |
| 59 | Sleep | Total time between midnight and the end time of the main sleep (i.e., the longest sleep bout) | Night | All | 1 | -0.57 |
| 60 | Sleep | Total sleep records/ bouts | Evening | All | 2 | 0.51 |
| 61 | Steps | Minutes labeled by Fitbit as 'lightly active' | Morning | Weekend | 1 | 0.56 |
| 62 | Steps | Total number of steps | All | Weekday | 1 | 0.27 |
| 63 | Steps | Minutes labeled by Fitbit as 'fairly active' | Afternoon | Weekday | 2 | 0.24 |
| 64 | Steps | Minutes labeled by Fitbit as 'sedentary' | Night | Weekday | 3 | 0.2 |
| 65 | Steps | Minutes labeled by Fitbit as 'lightly active' | All | Weekend | 1 | 0.19 |
| 66 | Steps | Total number of steps | All | All | 1 | 0.19 |
| 67 | Steps | Total number of steps | Night | Weekday | 5 | 0.18 |
| 68 | Steps | Minutes labeled by Fitbit as 'very active' | All | All | 2 | 0.18 |
| 69 | Steps | Minutes labeled by Fitbit as 'very active' | Afternoon | Weekday | 1 | 0.15 |
| 70 | Steps | Minutes labeled by Fitbit as 'sedentary' | Morning | Weekend | 2 | 0.12 |
| 71 | Steps | Minutes labeled by Fitbit as 'fairly active' | Evening | All | 1 | 0.12 |
| 72 | Steps | Minutes labeled by Fitbit as 'very active' | All | Weekday | 1 | 0.12 |
| 73 | Steps | Total number of steps | All | Weekend | 1 | 0.11 |
| 74 | Steps | Total number of steps | Afternoon | Weekend | 1 | 0.11 |
| 75 | Steps | Minutes labeled by Fitbit as 'very active' | Night | All | 2 | 0.11 |
| 76 | Steps | Minutes labeled by Fitbit as 'lightly active' | Afternoon | Weekend | 1 | 0.11 |
| 77 | Steps | Minutes labeled by Fitbit as 'very active' | Morning | All | 1 | 0.11 |
| 78 | Steps | Minutes labeled by Fitbit as 'fairly active' | Evening | Weekend | 1 | 0.07 |
| 79 | Steps | Minutes labeled by Fitbit as 'sedentary' | Night | All | 1 | 0.07 |

### C. Data Availability

Code for analysis is available at https://github.com/prernaa/MSCOVIDPaperCode. Summary and anonymous portion of the data for this study are available upon reasonable request to the corresponding author. Given the challenge of fully anonymizing sensor data, particularly the global positioning system tracking, sharing of full data set with external investigators will require IRB approval by the institution that governs the investigator making the request and institution-approved data transfer agreement.
